# Sampling strategies and integrated reconstruction for reducing distortion and boundary slice aliasing in high‐resolution 3D diffusion MRI

**DOI:** 10.1002/mrm.29741

**Published:** 2023-06-15

**Authors:** Ziyu Li, Karla L. Miller, Jesper L. R. Andersson, Jieying Zhang, Simin Liu, Hua Guo, Wenchuan Wu

**Affiliations:** ^1^ Wellcome Centre for Integrative Neuroimaging, FMRIB, Nuffield Department of Clinical Neurosciences University of Oxford Oxford UK; ^2^ Center for Biomedical Imaging Research, Department of Biomedical Engineering, School of Medicine Tsinghua University Beijing China

**Keywords:** 3D multi‐slab imaging, blip‐reversed acquisition, CAIPI, SPIRiT reconstruction, tractography

## Abstract

**Purpose:**

To develop a new method for high‐fidelity, high‐resolution 3D multi‐slab diffusion MRI with minimal distortion and boundary slice aliasing.

**Methods:**

Our method modifies 3D multi‐slab imaging to integrate blip‐reversed acquisitions for distortion correction and oversampling in the slice direction (k_z_) for reducing boundary slice aliasing. Our aim is to achieve robust acceleration to keep the scan time the same as conventional 3D multi‐slab acquisitions, in which data are acquired with a single direction of blip traversal and without k_z_‐oversampling. We employ a two‐stage reconstruction. In the first stage, the blip‐up/down images are respectively reconstructed and analyzed to produce a field map for each diffusion direction. In the second stage, the blip‐reversed data and the field map are incorporated into a joint reconstruction to produce images that are corrected for distortion and boundary slice aliasing.

**Results:**

We conducted experiments at 7T in six healthy subjects. Stage 1 reconstruction produces images from highly under‐sampled data (*R* = 7.2) with sufficient quality to provide accurate field map estimation. Stage 2 joint reconstruction substantially reduces distortion artifacts with comparable quality to fully‐sampled blip‐reversed results (2.4× scan time). Whole‐brain in‐vivo results acquired at 1.22 mm and 1.05 mm isotropic resolutions demonstrate improved anatomical fidelity compared to conventional 3D multi‐slab imaging. Data demonstrate good reliability and reproducibility of the proposed method over multiple subjects.

**Conclusion:**

The proposed acquisition and reconstruction framework provide major reductions in distortion and boundary slice aliasing for 3D multi‐slab diffusion MRI without increasing the scan time, which can potentially produce high‐quality, high‐resolution diffusion MRI.

## 1 INTRODUCTION

Diffusion MRI probes tissue at the microscopic scale non‐invasively,^1^, ^2^ providing information about healthy and pathological changes to neural architecture. High‐resolution diffusion MRI can depict microstructure details in the brain, facilitating tracking of thin fibers and accurate depiction of complex fiber configurations.^3^, ^4^, ^5^, ^6^ Because of these advantages, high‐resolution diffusion MRI is compelling for neuroscientific research and clinical diagnosis.

The 3D multi‐slab acquisition has great potential to achieve high‐resolution in‐vivo diffusion MRI, which can produce optimal SNR efficiency for spin‐echo‐based diffusion MRI due to its compatibility with a short TR = 1–2 s and achieve thin slices using a 3D k‐space encoding.^7^, ^8^, ^9^, ^10^, ^11^ 3D multi‐slab acquisitions divide the whole imaging volume into multiple thin slabs (typically with 10–20 slices in each slab) and encode each slab with a 3D k‐space readout, typically using a 3D EPI trajectory for an efficient acquisition.^7^, ^8^, ^9^ However, the image quality of 3D multi‐slab imaging with EPI‐based trajectories is compromised by two major image artifacts: distortion and boundary slice aliasing.

First, similar to conventional 2D EPI, 3D EPI suffers from distortions from the static (B_0_) magnetic field inhomogeneity.^12^ These distortions occur along the phase‐encoding direction due to its low bandwidth, especially near tissue/bone and tissue/air interfaces with large susceptibility differences. EPI distortions are conventionally corrected after image reconstruction using a field map acquired from a separate scan, using either a GRE field mapping sequence^12^ or a pair of EPI images acquired with opposite phase‐encoding directions.^13^ However, field mapping‐based corrections are inadequate, particularly if distortions become sufficiently severe that the signal from multiple voxels overlap in the reconstructed image. Moreover, static field maps cannot capture dynamic B_0_ field changes due to subject motion, eddy currents, and field drift across different diffusion directions.^14^

EPI distortion corrections using a pair of blip‐reversed phase‐encoding images have also been developed, which have demonstrated superior performance compared to the field mapping‐based correction in diffusion MRI.^13^, ^15^, ^16^, ^17^, ^18^, ^19^ While most of these approaches perform distortion correction using separately reconstructed blip‐up and blip‐down images,^13^, ^15^, ^16^ methods that jointly reconstruct blip‐up and blip‐down data have also been developed.^17^, ^18^, ^19^ The recently proposed BUDA (blip‐up/down acquisition) EPI^18^ method performed interleaved blip‐reversed acquisitions for each diffusion direction and incorporated field maps estimated from separate blip‐up/down images into a distortion corrected joint reconstruction. The method demonstrated improved distortion correction, dynamic B_0_ field mapping capability, and reduced g‐factor penalty due to the combination of blip‐up/down data in the joint reconstruction. However, existing blip‐reversed acquisition methods require doubled scan time compared with conventional single‐shot EPI using a single phase‐encoding acquisition.

A second source of artifacts in 3D multi‐slab imaging is slice aliasing near the slab boundary. Boundary slice aliasing arises due to the inability to achieve a sharp excitation profile, which means some tissue beyond the targeted slab is excited. The transition bands and sidelobes of the RF profile thus extend to adjacent slabs, leading to aliasing of signal from one end of the slab into the other.^20^ To avoid aliasing, conventional methods expand the FOV along the slice direction for each slab via oversampling^9^ or increase the overlapping between adjacent slabs.^9^ However, these methods inevitably require additional scan time.

In this work, we aim to incorporate the blip‐reversed acquisition and kz oversampling into 3D multi‐slab diffusion imaging to minimize distortion and boundary slice aliasing without increasing the scan time. However, achieving this goal is fundamentally challenging. Acquiring both blip‐up and blip‐down images without increasing the scan time requires an extra two‐fold (2×) acceleration for time compensation. The current 3D multi‐slab diffusion MRI acquisition typically uses 3D multi‐shot EPI with each EPI readout covering a single k_z_ plane. Because ˜50% of sequence time is dedicated to diffusion preparation, shortening the readout with in‐plane acceleration will not provide significant scan time reduction. Instead, the most effective way to reduce scan time is to accelerate along the slice direction, which determines the number of excitations required to sample along k_z_. This is challenging for in‐vivo diffusion MRI because each slab needs to be sufficiently thin (i.e., 10–20 mm) to ensure that the motion‐induced phase variation within each slab can be accurately measured with a 2D navigator,^9^, ^21^, ^22^ which leads to very limited coil sensitivity variation along the slice direction. Therefore, direct under‐sampling along the slice direction will result in a highly ill‐posed reconstruction. Moreover, to additionally address the boundary slice aliasing, oversampling along k_z_ is needed, which will lead to an even higher acceleration factor and thus a more challenging reconstruction problem.

**FIGURE 1:**
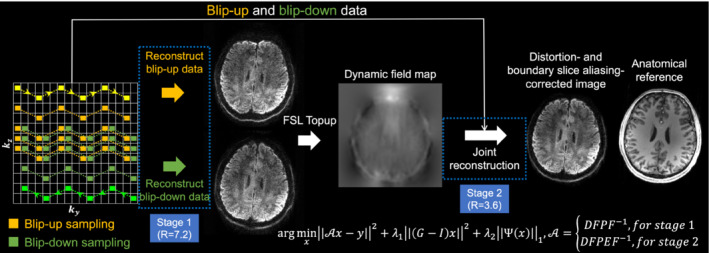
Proposed framework. The proposed sampling pattern with k_z_ blipped‐CAIPI and complementary partial Fourier for blip‐up (yellow) and blip‐down (green) data and the two‐stage reconstruction. The trajectory of one shot of the multi‐shot sampling is marked in bright yellow and bright green for blip‐up and blip‐down sampling with arrows indicating the phase encoding direction. The forward operator A in the reconstruction includes Fourier transform F and inverse Fourier transform F^‐1^, the phase modulation P representing motion‐induced phase errors measured by 2D navigators, k‐space sampling operation D in both stages 1 and 2, and an additional distortion operation E (captured by the field map) for stage 2. Example images are from a single volume diffusion MRI dataset (1.05 mm isotropic resolution) of a representative subject, with anatomical image listed for reference (acquired with MPRAGE at 0.86 mm isotropic resolution).

We propose an approach for 3D EPI that enables distortion‐ and boundary slice aliasing‐corrected reconstruction similar to BUDA^18^ but with no increase in scan time compared to the conventional 3D multi‐slab acquisition^7^, ^8^, ^9^ (Figure 1). We acquire an equal number of shots with blip‐up and blip‐down phase encoding. The reconstruction proceeds in two stages. In the first stage, we reconstruct the highly under‐sampled blip‐up and blip‐down images separately in order to estimate the field map.^13^, ^23^, ^24^ In the second stage, we reconstruct a final image that is distortion corrected and has a more modest acceleration using all segments and accounting for the field map. In addition, we enlarge the FOV along the slice direction to reduce the boundary slice aliasing. This approach relies on the highly‐accelerated reconstruction for stage 1 being sufficiently robust to accurately estimate the field map. To support this aim, we use a 3D EPI trajectory with blipped CAIPI^25^ and partial Fourier along k_z_. A high‐SNR SPIRiT‐based^26^ reconstruction is also optimized and employed. Our framework is validated with in‐vivo experiments on a 7T scanner and achieves whole‐brain diffusion imaging at 1.05 mm isotropic resolution. The diffusion analysis and tractography results reveal the higher anatomical fidelity and quantitative accuracy of the proposed method compared to the conventional 3D multi‐slab imaging, demonstrating the potential of our method to facilitate high‐resolution diffusion MRI with improved image quality to benefit neuroscientific research.

## 2 METHODS

### 2.1 Blip‐reversed acquisition and reconstruction for 3D multi‐slab imaging

#### 2.1.1 Acquisition

**FIGURE 2:**
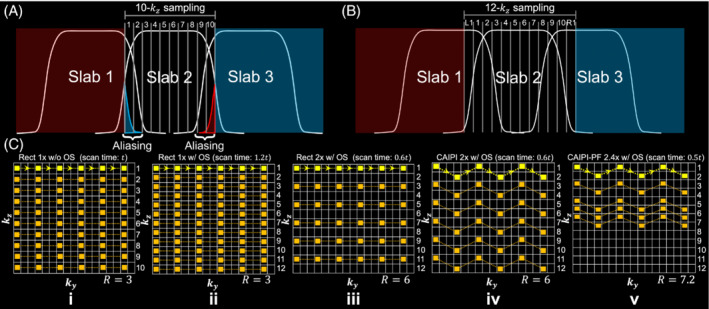
3D multi‐slab acquisition and sampling patterns for blip‐up data. (A) Demonstration of slice aliasing at slab boundary caused by non‐rectangular RF profile and limited FOV along the slice direction for 3D multi‐slab acquisition (10‐kz sampling). (B) Correction of slice aliasing by expanding the FOV for each slab through k_z_ over‐sampling (12‐k_z_ sampling) (L1 and R1 denote the 1 oversampled slice on the left and right side). (C) Comparison of various sampling patterns: rectangular sampling without oversampling along k_z_ (Rect 1× w/o OS) (i); rectangular sampling with 20% oversampling along k_z_ (Rect 1× w/OS) (ii); rectangular sampling with 20% oversampling and 2× acceleration along k_z_ (Rect 2× w/OS) (iii); CAIPI sampling with 20% oversampling and 2× acceleration along k_z_ (CAIPI 2× w/OS) (iv); CAIPI‐PF sampling with 20% oversampling, partial Fourier and 2.4× acceleration along k_z_ (CAIPI‐PF 2.4× w/ OS) (v). A 3× acceleration along k_y_ is applied in all sampling patterns. The trajectory of one EPI shot is marked in bright yellow with arrows indicating the phase encoding direction. The parameter *t* represents the scan time of the acquisition without oversampling or acceleration along k_z_ for one phase encoding direction (C, i). *R* is the total under‐sampling factor for each sampling pattern.

Integrating 3D multi‐slab diffusion MRI with blip‐reversed EPI requires the acquisition of two 3D EPI images with reversed phase encodings, which can be achieved at a cost of doubled scan time. One approach to shorten the scan time is to perform under‐sampling along k_z_. By acquiring half the segments with blip‐up phase encoding and the other half with blip‐down phase encoding, the total scan time of the integrated blip‐reversed acquisition is identical to conventional 3D multi‐slab EPI.^7^, ^8^, ^9^ Importantly, 3D multi‐slab diffusion MRI requires very thin slabs to facilitate the correction of motion‐induced errors.^8^, ^9^, ^22^ This severely limits the variation of coil profiles along the slab direction, and as a result conventional rectangular under‐sampling with integer reduction along k_y_ and k_z_ (e.g., Figure 2C,iii) may suffer from a high g‐factor penalty and residual aliasing.

Moreover, due to the limited FOV and non‐rectangular RF profile along the slice direction, where transition bands and side lobes extend to adjacent slabs (Figure 2A), slice aliasing happens at slab boundaries in 3D multi‐slab imaging. The slice aliasing artifacts can be reduced by over‐sampling along k_z_ with an extended FOV (Figure 2B). To extend the FOV without increasing the number of shots (e.g., from 10 shots to 12 shots comparing Figure 2C,i,ii), a higher acceleration factor along k_z_ is required, which leads to a more challenging reconstruction.

Here, we combined blip‐reversed acquisitions with two sampling strategies to improve the under‐sampled reconstruction, which is particularly important for the first stage of reconstruction in which images are separately reconstructed for the blip‐up and blip‐down segments. First, k_z_ blipped‐CAIPI^25^, ^27^ was integrated for more effective use of coil sensitivity, where each shot covers the full extent of the phase encoding direction (k_y_) with even spacing along k_y_ and includes “blips” to traverse closely spaced k_z_ planes (Figure 2C,iv). We also employed partial Fourier along k_z_ (Figure 2C,v), which reduces aliasing in the slice direction through a more densely sampled central k‐space region. This achieves an even shorter scan time by reducing the number of shots (Figure 2C,v, halved scan time compared to Figure 2C,i). The proposed sampling approach enables simultaneous correction of distortions (by allowing joint acquisition of blip‐up/down data) and boundary slice aliasing (by oversampling along k_z_) without increasing the scan time. We refer to our sampling approach as “CAIPI‐PF” hereafter.

For the joint acquisition of blip‐reversed data, equal numbers of shots traversing k_y_ in opposite directions are acquired. The blip‐up and blip‐down sampling cover the complementary subsets of the k‐space with a shift of $∆k_{y}$ and complementary partial Fourier along k_z_ (Figure 1, left), which reduces the noise amplification and prevents resolution loss in the stage 2 joint reconstruction.

#### 2.1.2 Reconstruction

The cost function of the SPIRiT‐based regularized reconstruction is:

(1) $${\left\|Ax-y\right\|}_{2}^{2}+\lambda_{1}{\left\|\left(G-I\right)x\right\|}_{2}^{2}+\lambda_{2}{\left\|\Psi \left(x\right)\right\|}_{1}$$

where *x* is the multi‐coil k‐space data of the target aliasing‐ and distortion‐corrected image, A is the forward operator, *y* is the acquired data, *G* is the SPIRiT kernel trained on coil calibration data, *I* is the identity matrix, Ψ is the wavelet operator, and $\lambda_{1}$ and $\lambda_{2}$ are the parameters for the SPIRiT and sparsity regularizations (the SPIRiT weights for stage 1 and stage 2 are denoted as $\lambda_{1,1}$ and $\lambda_{1,2}$, respectively). The SPIRiT regularization ($\lambda_{1}$) facilitates parallel imaging by enforcing calibration consistency between every k space data point and its neighbors,^26^ while the sparsity regularization ($\lambda_{2}$) is used to suppress the noise.^28^

The forward operator A is constructed differently for the two stages:

(2) $$A=\left\{\begin{array}{ll} \mathrm{DFP}F^{-1},\; & \text{for stage}\;1\\ \text{DFPE}F^{-1},\; & \text{for stage}\;2 \end{array}\right.$$

where *D* is the down‐sampling operator in k‐space, F and $F^{-1}$ are the Fourier transform and inverse Fourier transform, respectively, and *P* represents motion‐induced phase errors captured by 2D navigators. The *E* operator is only applied in stage 2 and represents spatial distortion induced by field inhomogeneity estimated from the stage 1 reconstruction.

It is worth noting that unlike the original SPIRiT formulation^26^ in which data consistency can be ensured implicitly by only estimating the missing k‐space points, in our work the entire k‐space needs to be estimated. This is because, in our forward operator A, the acquired k‐space points are corrupted by the motion‐induced phase *P* and the distortion‐induced displacement *E* and are, therefore, different from those in the uncorrupted target k‐space data *x*. Hence, the SPIRiT constraint is explicitly added as a regularization term and the data consistency term ${\left\|Ax-y\right\|}_{2}^{2}$ imposes the consistency constraint across all k‐space points.

### 2.2 In‐vivo experiments

A 3D multi‐slab spin‐echo diffusion MRI sequence^8^ was modified to integrate blipped‐CAIPI and kz partial Fourier sampling. After the imaging echo, a second refocusing pulse was applied to acquire a low‐resolution 2D navigator to correct the motion‐induced phase errors. The sequence diagram of the proposed acquisition is demonstrated in Figure S1. Subjects were scanned on a Siemens 7T scanner using a 32‐channel receive coil. Written informed consent in accordance with local ethics was obtained from each subject.

#### 2.2.1 Evaluation protocols

To evaluate the impact of different sampling patterns and reconstruction parameters, fully sampled single‐slab datasets using CAIPI sampling were acquired from a single subject using the following scan parameters: 1.22 mm isotropic resolution, 20 slices, TE1(imaging)/TE2(navigator)/TR = 72/128/1800 ms, *b* = 1000s/mm^2^, diffusion encoding along left–right direction, k_y_ under‐sampling R_y_ = 3, echo spacing 0.76 ms. The 2D navigator acquired 64 phase encoding lines using the same phase‐encoding direction, the same k_y_ under‐sampling factor, and the same echo spacing as the imaging echo. An illustration of the navigator acquisition trajectory is showed in Figure S2. To avoid slice aliasing, 1.2× oversampling along k_z_ was applied, which encoded a larger FOV of 29.3 mm along the slice direction with 24 slices. Blip‐up and blip‐down phase encoding datasets were acquired separately. Three scans with 0, 1, and 2 $∆k_{y}$ shift were acquired and combined to produce fully sampled data. In addition, a dataset using conventional rectangular sampling without k_z_ oversampling was also acquired from the same subject to demonstrate slice aliasing.

Retrospective under‐sampling was then performed to evaluate different sampling patterns, which were similar to those shown in Figure 2 but with 20 or 24 kz encoding planes (without or with kz oversampling). For CAIPI‐PF sampling (Figure 2C,v), the shots #1,3,5,7,9,11,12,13,14,15 were used for acquiring blip‐up data and the shots #11,12,13,14,15,16,18,20,22,24 were used for acquiring blip‐down data (shot #*n* covers k_z_ plane *n* and *n* + 1) (partial Fourier factor: $f_{\mathrm{pf}}=2/3$).

For whole‐brain quantitative comparison of the proposed CAIPI‐PF sampling (Figure 2C,v) with the conventional rectangular sampling (Figure 2C,i), three subjects were scanned using 1.22 mm isotropic resolution reference protocol, CAIPI‐PF sampling protocol, and conventional sampling protocol. For all protocols, six slabs with 20 slices per slab were acquired. Neighboring slabs were overlapped by 1 slice, resulting in 115 slices in the final reconstruction. The FOV was 220 × 220 × 140 mm^3^ and voxel size was 1.22 mm isotropic. Interleaved slab acquisition was used to minimize cross talk between adjacent slabs. Diffusion‐weighted images were acquired with *b* = 1000s/mm^2^ and 16 diffusion directions uniformly sampled on a sphere. The echo spacing was 0.78 ms, and *R*_*y*_ = 3 acceleration was applied along ky phase encoding, resulting in an effective echo spacing of 0.26 ms. TE1(imaging)/TE2(navigator)/TR = 73/130/1800 ms. For the reference protocol, the blip‐up and blip‐down diffusion weighted datasets were separately acquired with rectangular sampling with no acceleration and 20% oversampling along k_z_ (as in Figure 2C,ii), with a scan time of ˜25 min. For the CAIPI‐PF imaging protocol, the diffusion weighted data were acquired using the CAIPI‐PF sampling (i.e., Figure 2C,v) ($f_{\mathrm{pf}}=2/3$) with 10 blip‐up shots and 10 blip‐down shots for each slab. The acquisitions of blip‐up and blip‐down shots were consecutive within each diffusion encoding direction. For the conventional imaging protocol, the diffusion weighted data were acquired using 20 k_z_ rectangular sampling (Figure 2C,i). The scan times for the CAIPI‐PF and conventional imaging protocols were both ˜10.5 min. One set of *b* = 0 image was acquired with k_z_ fully sampled (i.e., 24 blip‐up shots and 24 blip‐down shots for each slab) and used for the diffusion analyses of all protocols, with a scan time of ˜1.4 min.

#### 2.2.2 High‐resolution diffusion protocols

Six subjects were scanned using a 1.05 mm isotropic resolution CAIPI‐PF imaging protocol to demonstrate the robustness of the proposed method. Diffusion‐weighted images were acquired with *b* = 1000s/mm^2^ and 48 diffusion directions uniformly sampled on a sphere with interleaved 3 *b* = 0 image volumes. The echo spacing was 0.82 ms, and *R*_*y*_ = 3 acceleration was applied along k_y_ phase encoding, resulting in an effective echo spacing of 0.27 ms. TE1(imaging)/TE2(navigator)/TR = 82/150/1800 ms. The FOV was 220 × 220 × 121 mm^3^ and voxel size was 1.05 mm isotropic. The diffusion weighted data were acquired using the same CAIPI‐PF sampling as in the evaluation protocol (i.e., Figure 2C,v). To reduce aliasing artifacts from CSF signal, *b* = 0 images were acquired with k_z_ fully sampled (i.e., 24 blip‐up shots and 24 blip‐down shots for each slab). The total scan time was ˜33 min (36 s per diffusion direction).

In one subject, a dataset using a conventional 3D multi‐slab high‐resolution protocol^8^ was also acquired using 20 k_z_ rectangular sampling (Figure 2C,i) to enable comparisons at 1.05 mm isotropic resolutions. Eight *b* = 0 images, including six blip‐up and two blip‐down volumes, were interspersed into the diffusion‐weighted image acquisition. Other scan parameters were the same as the CAIPI‐PF protocol and the total scan time was ∼34 min.

An MPRAGE image was also acquired for each subject as an anatomical reference (˜5 min) at 0.86 mm isotropic resolution.

### 2.3 Reconstruction details

The integrated blip‐reversed 3D multi‐slab EPI data were reconstructed using the proposed two‐stage reconstruction, while the conventional 3D multi‐slab EPI data and reference separate blip‐up and blip‐down data were reconstructed by the stage 1 reconstruction. The SPIRiT kernel used in the reconstruction was trained using gradient echo coil calibration data. The image reconstruction was conducted offline in MATLAB. Image processing were conducted using functions from FMRIB Software Library (FSL)^23^ unless indicated otherwise. Equation 1 was optimized with preconditioned conjugate gradient method with variable splitting.^29^ The k‐space data were first Fourier transformed along k_x_ followed by reconstruction performed for each $k_{y}-k_{z}$ plane using a 5 × 5 SPIRiT kernel. The reconstructed 2D images were concatenated along the readout direction (*x*) to form the whole image volume. The 32‐channel data were compressed to 8 to shorten the calculation time.^30^ The field map was calculated using “topup”.^13^, ^24^ On a 2.9 GHz Quad‐Core Intel Core i7 CPU, the computation time for one $k_{y}-k_{z}$ plane of one slab is ˜15 s for stage 1 reconstruction, and ˜90 s for stage 2 reconstruction. The processing time for “topup” is ˜5 min per slab.

In stage 1 reconstruction, the unacquired partial Fourier region was zero filled before inverse Fourier transform along k_z_, resulting in smoothing along the slice direction. As the ΔB_0_ field is spatially smooth and the slab is thin, the impact of slice smoothness on field map estimation with “topup” is expected to be not significant. No zero filling is necessary for the stage 2 reconstruction because it operates on the full k_z_ extent (complementary blip‐up and blip‐down segments).

The 2D navigator images were reconstructed with 2D GRAPPA^31^ using a 2D calibration dataset, which was acquired separately for blip‐up and blip‐down data.^8^ The phase images were extracted and used as an estimation of motion induced phase errors.

Based on the balance between the data consistency and the regularization effect of SPIRiT, we empirically searched the appropriate weights at different orders of magnitude (i.e., $\lambda_{1,1}$ = 0, 0.1, 1, 10 and $\lambda_{1,2}$ = 0, 1, 10, 100 for stage 1 and stage 2, respectively). The weights that produce the lowest NRMSE were selected (i.e., $\lambda_{1,1}=1$ and $\lambda_{1,2}=10$) and further fine‐tuned to obtain the final weights for reconstruction (i.e., $\lambda_{1,1}=1$ and $\lambda_{1,2}=20$) (Figure S3). $\lambda_{1,1}=1$ also produces results with the most accurate field map estimation (Figure S4). These optimal weights were used for the reconstruction of the whole‐brain data from the evaluation and high‐resolution protocols. The sparsity regularization parameter $\lambda_{2}=0.7\times 10^{-3}$ was used in both stage 1 and stage 2 reconstructions. The estimated field maps were compared with reference field maps derived from fully sampled blip‐up and blip‐down images, and voxel displacement errors were calculated by multiplying the field map difference (in Hz) with the readout duration (in seconds). NRMSE and mean voxel displacement errors were calculated within a brain mask. The reconstruction codes are openly availiable at https://github.com/liziyu0929/distortion‐free‐3d‐diffusion‐mri.

The g‐factors for different blip‐up sampling patterns demonstrated in Figure 2 were evaluated, which were calculated using the pseudo‐multiple replica method^32^ with 100 repetitions of the Monte‐Carlo simulation. In each Monte‐Carlo repetition, independent and identically distributed complex Gaussian noise was added to the pre‐whitened multi‐channel k‐space data. For the stage 1 CAIPI‐PF sampling, the impact of k_z_ partial Fourier on g‐factor was accounted by dividing the resultant g‐factor with the square root of partial Fourier factor as suggested by Kettinger et al.^33^ The sparsity constraint was not included in g‐factor calculations to reduce non‐linearity. The g‐factors were calculated within a brain mask.

### 2.4 Post‐processing

Slab combination and correction for slab saturation artifacts were performed using “NPEN”.^20^ The whole‐brain images were corrected for Gibbs ringing using “mrdegibbs3D” (https://github.com/jdtournier/mrdegibbs3D).^34^, ^35^ For the reference data acquired with the evaluation protocol, the whole‐brain blip‐up and blip‐down data were processed by “topup” to obtain the distortion corrected images for each diffusion direction. The distortion corrected images were then processed with “eddy” without “‐topup” option. For conventional 3D multi‐slab data acquired with the evaluation and high‐resolution protocols, a whole‐brain field map was estimated using blip‐up and blip‐down *b* = 0 image volumes using “topup”, which was then input to “eddy”^36^ along with all diffusion data to correct for susceptibility and eddy current induced distortions. The CAIPI‐PF data acquired with the evaluation and high‐resolution protocols were processed with “eddy” without “‐topup” option, as field inhomogeneity‐induced distortion has been corrected in the reconstruction. To evaluate the efficacy of the proposed method in estimating diffusion direction‐dependent dynamic field maps and reducing the eddy current induced geometric distortions, the high‐resolution CAIPI‐PF data and conventional 3D multi‐slab data were also processed by “eddy” without eddy current correction, which was facilitated by specifying “—flm = movement” in the command line of “eddy”.

### 2.5 Diffusion analysis

All diffusion analyses were performed in the native diffusion space. For the evaluation protocol, as the diffusion data from the different methods were acquired in the same scan and were all co‐aligned with the same *b* = 0 image, they were already in the same native diffusion space. Diffusion tensor model fitting was performed on the whole‐brain diffusion data for quantitative comparison between different methods.

For the high‐resolution diffusion protocols, one subject was scanned twice using the CAIPI‐PF and conventional 3D multi‐slab high‐resolution protocol, whose results were co‐registered to the anatomical space for comparison. These datasets were acquired in separate sessions due to the long scan times. The diffusion data from both samplings were co‐registered to the MPRAGE image. Due to different head positions, distortions induced by gradient nonlinearity were different between the two scans. For fair comparison, the MPRAGE image was acquired in a separate session along with an intermediate *b* = 0 image volume with matched gradient nonlinearity distortions to minimize the impact of gradient nonlinearity and improve the co‐registration accuracy. These co‐registration steps are listed in the Co‐registering Details section in Supplementary Information.

For the remaining subjects, T1w images were co‐registered to the diffusion space. The *b* = 0 images were co‐registered to the T1w images using “epi_reg”.^37^, ^38^ The resultant transformations were then inverted using “convert_xfm”^37^, ^38^ and applied to the T1w images.

The diffusion tensor model fitting was performed using “dtifit”.^24^ White matter tractography was performed using “autoPtx”,^39^, ^40^, ^41^ which includes the pre‐processing stage that runs probabilistic model fit using “bedpostx”,^40^ and the tractography stage which runs the probabilistic tractography using “probtrackx”.^40^

## 3 RESULTS

**FIGURE 3:**
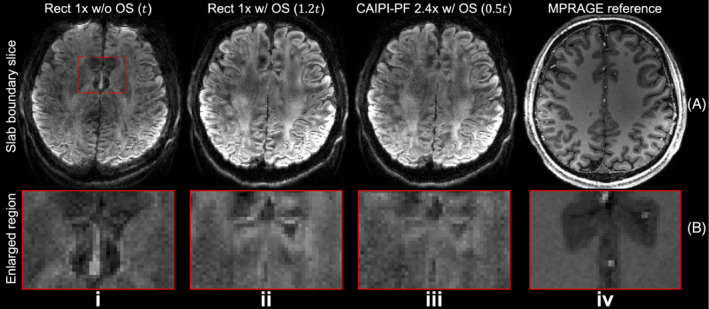
Boundary slice aliasing correction. The boundary slices (A) and enlarged regions (B) of blip‐up data from rectangular sampling without oversampling along k_z_ (*R*_*z*_/*R*_*y*_ = 1/3) (i), rectangular sampling with 20% oversampling and 2× acceleration along k_z_ (*R*_*z*_/*R*_*y*_ = 1/3) (ii), the proposed CAIPI‐PF sampling with 20% oversampling, partial Fourier and 2.4x acceleration along k_z_ (*R*_*z*_/*R*_*y*_ = 2.4/3) (iii) acquired with the evaluation protocol (1.22 mm isotropic resolution), and the reference MPRAGE data (0.86 mm isotropic resolution) (iv) are displayed, with relative scan times listed for each method except for MPRAGE reference. The parameter *t* represents the scan time of a rectangular sampling without over‐sampling or acceleration along k_z_ (as Figure 2C,i).

Figure 3 shows the effectiveness of the proposed sampling in reducing the slice aliasing in the slab boundary slice. Without oversampling, the boundary slice suffers from aliasing (comparing Figure 3,i with Figure 3,iv) caused by non‐rectangular RF profile and limited FOV (Figure 2A). Simple oversampling along k_z_ increases the FOV (Figure 2B) and corrects the aliasing (comparing Figure 3,ii with Figure 3,iv), but requires longer scan time. Our proposed CAIPI‐PF sampling produces boundary slice aliasing‐corrected images with a much faster acquisition, reducing scan time from 1.2 to 0.5 *t* (*t* represents the scan time of a rectangular sampling without over‐sampling or acceleration along k_z_, as Figure 2C,i).

**FIGURE 4:**
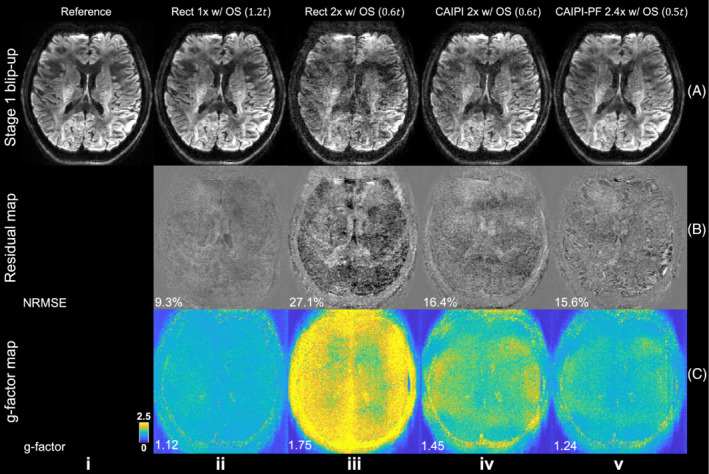
Stage 1 blip‐up reconstruction. The blip‐up data for a slice at slab center (A) from fully sampled reference (i) and different under‐sampling patterns: rectangular sampling with 20% oversampling along k_z_ (Rect 1× w/OS) (ii); rectangular sampling with 20% oversampling and 2× acceleration along k_z_ (Rect 2× w/OS) (iii); CAIPI sampling with 20% oversampling and 2× acceleration along k_z_ (CAIPI 2× w/OS) (iv); CAIPI‐PF sampling with 20% oversampling, partial Fourier and 2.4× acceleration along k_z_ (CAIPI‐PF 2.4× w/OS) (v), their residuals with the fully sampled reference (B), and their g‐factor maps (C) are displayed, with relative scan times listed for each method. The data were acquired with the evaluation protocol (1.22 mm isotropic resolution). The normalized RMS errors (NRMSE) and mean g‐factor of the whole slab are listed to quantify the image similarity and noise amplification. The parameter *t* represents the scan time of a rectangular sampling without over‐sampling or acceleration along k_z_ (as Figure 2C,i).

Results from stage 1 blip‐up reconstruction for different sampling patterns are demonstrated in Figure 4. The reference data were fully sampled with no acceleration along k_y_ or k_z_. The results with fully k_z_ encoding and *R*_*y*_ = 3 under‐sampling along k_y_ produces the most similar result to the reference and the lowest g‐factor (NRMSE: 9.3%; g‐factor: 1.12) with the longest acquisition time. Image from rectangular under‐sampling with *R*_*y*_/*R*_*z*_ = 3/2 suffers from strong aliasing due to the limited coil sensitivity variation along the thin slab, producing the highest error and g‐factor (NRMSE = 27.1%; g‐factor: 1.75). Using CAIPI sampling alone can substantially improve the reconstruction and lower the NRMSE (16.4%) and g‐factor (1.45) by more efficient use of coil sensitivity information. The partial Fourier strategy further reduces reconstruction errors with lower NRMSE (15.6%) and g‐factor (1.24) using an even shorter scan time. The blip‐down reconstruction produces consistent results with the blip‐up reconstruction (Figure S5).

**FIGURE 5:**
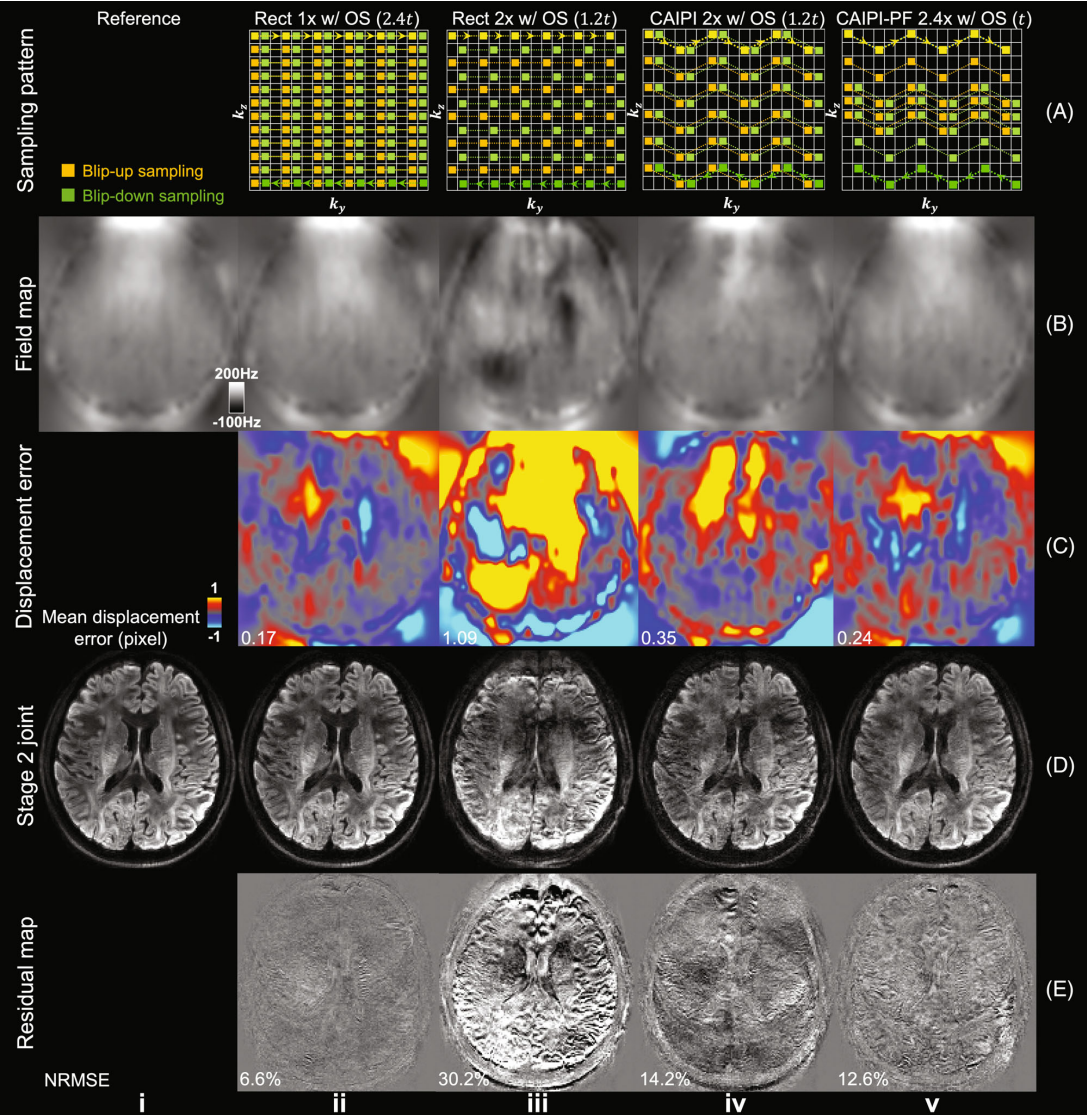
Field map estimation and joint reconstruction. The joint blip‐up (yellow) and blip‐down (green) sampling patterns (A), the corresponding estimated field maps from “topup” (B), the voxel displacement error maps (C), stage 2 joint reconstruction results (D), and residual maps (E) with the reference image acquired with the evaluation protocol (1.22 mm isotropic resolution) are displayed, with relative total scan times listed for each method. The parameter *t* represents the scan time of a rectangular sampling without over‐sampling or acceleration along k_z_ for one phase encoding direction (as Figure 2C,i). The trajectory of one shot of blip‐up and blip‐down sampling is marked in bright yellow and bright green, respectively. The mean displacement errors and normalized RMS errors (NRMSE) of the whole slab are listed to quantify the image similarity.

The estimated field maps and stage 2 joint reconstruction results for different joint blip‐reversed acquisition strategies are shown in Figure 5. The data acquired with full k_z_ sampling still produces the most accurate field map and the best stage 2 reconstruction (mean displacement error: 0.17 pixels; NRMSE: 6.6%). However, the scan time of this acquisition strategy is 2.4× the scan time of a conventional multi‐slab acquisition. In conventional rectangular under‐sampling, the reconstructed image exhibits obvious structural difference with the reference due to the inaccurate field map estimated from the aliased images from stage 1 reconstruction (mean displacement error: 1.09 pixels; NRMSE: 30.2%). The residual aliasing of regular CAIPI under‐sampling in stage 1 reconstruction results in errors in the estimated field map, which leads to inaccuracies in joint reconstruction results (mean displacement error: 0.35 pixels; NRMSE: 14.2%). The proposed CAIPI‐PF sampling provides an improved field map estimation and joint reconstruction with substantially better results due to a more densely sampled k‐space center (mean displacement error: 0.24 pixels; NRMSE: 12.6%) using the shortest scan time among all sampling patterns. The voxel displacement error map from CAIPI‐PF also shows similar spatial pattern to the fully k_z_‐sampled data without obvious anatomical bias, indicating the CAIPI‐PF sampling strategy does not introduce substantial errors in field map estimation.

**FIGURE 6:**
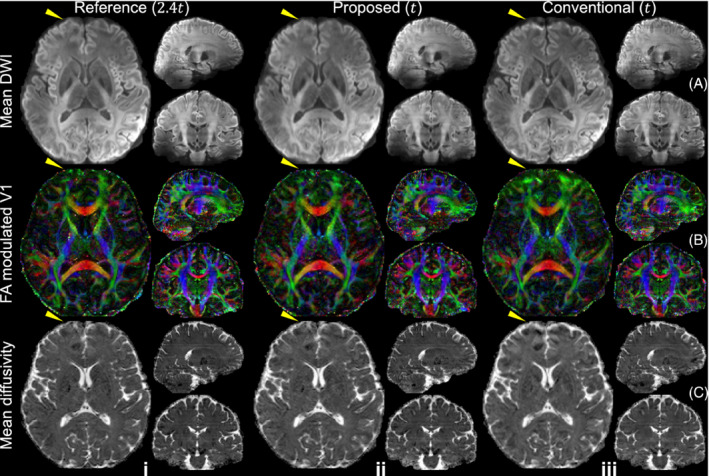
Comparisons of whole‐brain diffusion images. The mean diffusion‐weighted image (DWI) (A), the fractional anisotropy (FA) modulated primary eigenvector (V1) (B), and the mean diffusivity (C) of diffusion data acquired using blip‐up and blip‐down k_z_‐fully sampled (i.e., 24 blip‐up and 24 blip‐down shots for each diffusion direction) reference protocol (Reference, i), joint blip‐reversed with CAIPI‐PF protocol (Proposed, ii) and conventional 3D multi‐slab protocol using only blip‐up phase encoding (Conventional, iii) at 1.22 mm isotropic resolution from the same subject are displayed. The parameter *t* represents the scan time of a rectangular sampling without over‐sampling or acceleration along k_z_ for one phase encoding direction (as Figure 2C,i). The yellow arrows highlight the regions with strong distortions near frontal which cannot be fully corrected with the conventional method.

Figure 6 demonstrates the whole‐brain diffusion imaging results from the evaluation protocols of one representative subject. Overall, the 16‐direction DTI results exhibit high SNR thanks to the superior SNR efficiency provided by 3D multi‐slab imaging. However, limited distortion correction performance is observed on the results from the conventional 3D multi‐slab acquisition where diffusion weighted images were acquired with only blip‐up phase encoding, and distortion correction was applied using a field map derived from a pair of blip‐up/down *b* = 0 image, especially in the frontal region. The proposed CAIPI‐PF protocol produces results visually similar to the reference results acquired with 2.4× scan time. For quantitative comparison, the mean absolute differences of FA and MD compared to the reference results across the brain region (i.e., within a brain mask) were calculated for each subject. The group‐level means (± standard deviation) of the mean absolute differences of the fractional anisotropy (FA) (calculated within FA masks where FA >0.05) with the reference results are 0.0876 ± 0.00221 and 0.1004 ± 0.00170 for the proposed and conventional method, respectively. The mean absolute differences of mean diffusivity are 1.33 × 10^−4^ ± 6.49 × 10^−6^ mm^2^/s and 2.05 × 10^−4^ ± 2.29 × 10^−5^ mm^2^/s for the proposed and conventional method, respectively. The improved distortion and boundary slice‐aliasing correction performance of the proposed method contribute to its better quantitative accuracy.

**FIGURE 7:**
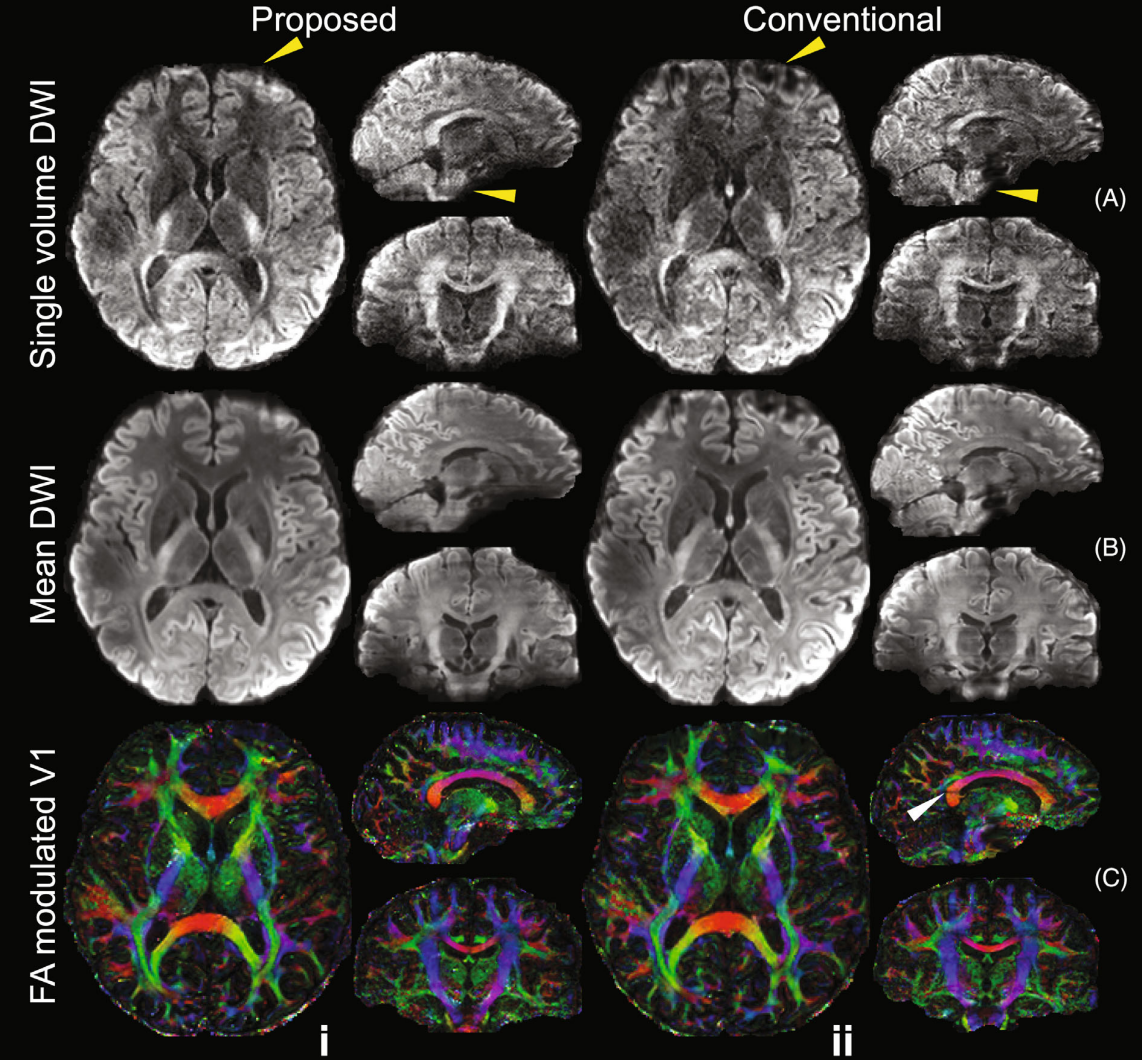
High‐resolution whole‐brain diffusion images. The single volume diffusion‐weighted images (DWI) along the diffusion direction (−0.10, 0.88, 0.47) (A), the mean DWI (B), and the fractional anisotropy (FA) modulated primary eigenvector (V1) (C) of diffusion data acquired using joint blip‐reversed with CAIPI‐PF sampling (Proposed, i) and conventional 3D multi‐slab sampling using only blip‐up phase encoding (Conventional, ii) at 1.05 mm isotropic resolution from the same subject are displayed. The diffusion data are co‐registered to the same anatomical data (0.86 mm isotropic resolution) for comparison.

Results from the 1.05 mm whole‐brain high‐resolution protocols are showed in Figure 7. Our proposed method produces single‐volume diffusion‐weighted image with high SNR and reduced artifacts and high‐quality whole‐brain DTI results. Compared to the conventional method, the proposed method achieves substantially improved anatomical fidelity, especially near the regions in the frontal lobe and pons where the field inhomogeneity is strong (Figure 7A, yellow arrows). The conventional method also suffers from residual slice aliasing at slab boundaries (Figure 7C,ii, the white arrow in the splenium of corpus callosum) due to the absence of over‐sampling and limited overlapping between slabs (only one slice overlapping) to match the scan time with the proposed method.

The alignment of the diffusion data with the anatomical reference is showed in Figure S6. When combining blip‐up and blip‐down data for distortion correction, the proposed method and the conventional method produce similar distortion correction on *b* = 0 images. However, the distortion‐corrected diffusion‐weighted images from the proposed method exhibits consistently improved anatomical fidelity for different diffusion directions. In addition, the proposed method can also correct eddy current induced distortions because the diffusion direction‐dependent dynamic field map is incorporated into the joint reconstruction (Figure S7). The co‐registered images with and without eddy current correction are highly similar from the proposed reconstruction, with a low mean NRMSE across all diffusion directions (1.7%), indicating most eddy current induced distortions have already been corrected during the reconstruction. In comparison, images from the conventional method exhibit larger differences before and after the eddy current correction, with a much higher mean NRMSE for all diffusion directions (9.4%).

**FIGURE 8:**
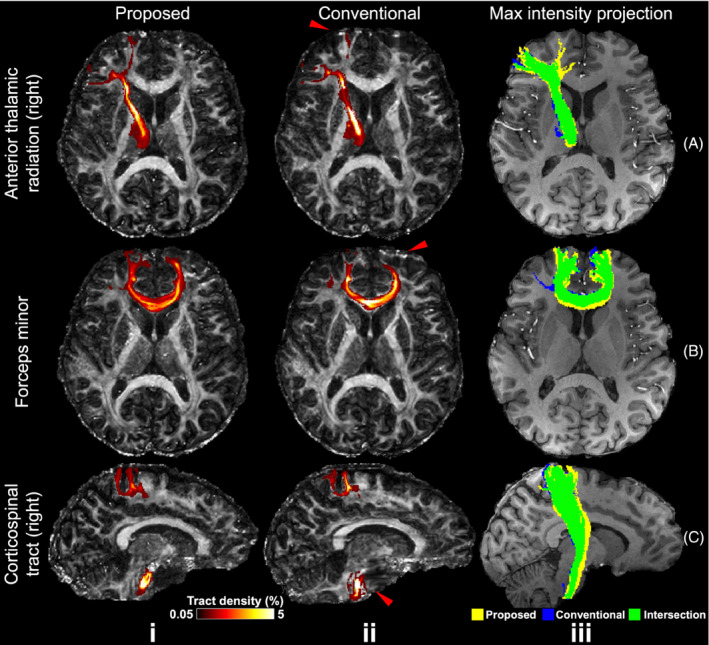
Comparison of tractography results. Tractography results of right anterior thalamic radiation (A), forces minor (B), right corticospinal tract (C) from the same subject using the proposed joint blip‐reversed with CAIPI‐PF (Proposed, i) and conventional 3D multi‐slab sampling (Conventional, ii) with high‐resolution protocols (1.05 mm isotropic) overlayed on their fractional anisotropy (FA) maps (tract density displaying range: 0.05%–5%), and their maximum intensity projection masks (iii) (binarized with tract density threshold: 0.3%) overlayed on the anatomical image. The red arrows highlight the regions with strong distortions near frontal (A, B, ii) and pons (C, ii), which cannot be fully corrected with the conventional method.

Tractography results show consistent improvement of the proposed method compared to the conventional method. Improvements can be seen in frontal regions with residual distortion for the anterior thalamic radiation (Figure 8A) and forceps minor (Figure 8B), where the proposed method captures projections into the cortical gray matter more successfully. Similarly, the corticospinal tracts are represented as thicker white matter bundles in tractography based on the proposed reconstruction compared to the conventional method, whose thickness is considerably reduced near the pons (red arrows, Figure 8C). The tract mask (binarized with tract density threshold: 0.3%) volumes from the proposed method increase by 20.2%, 43.0%, and 49.5% compared to those from the conventional method for right anterior thalamic radiation, forces minor, and right corticospinal tracts, respectively. The detailed tract mask volumes can be found in Table S1.

**FIGURE 9:**
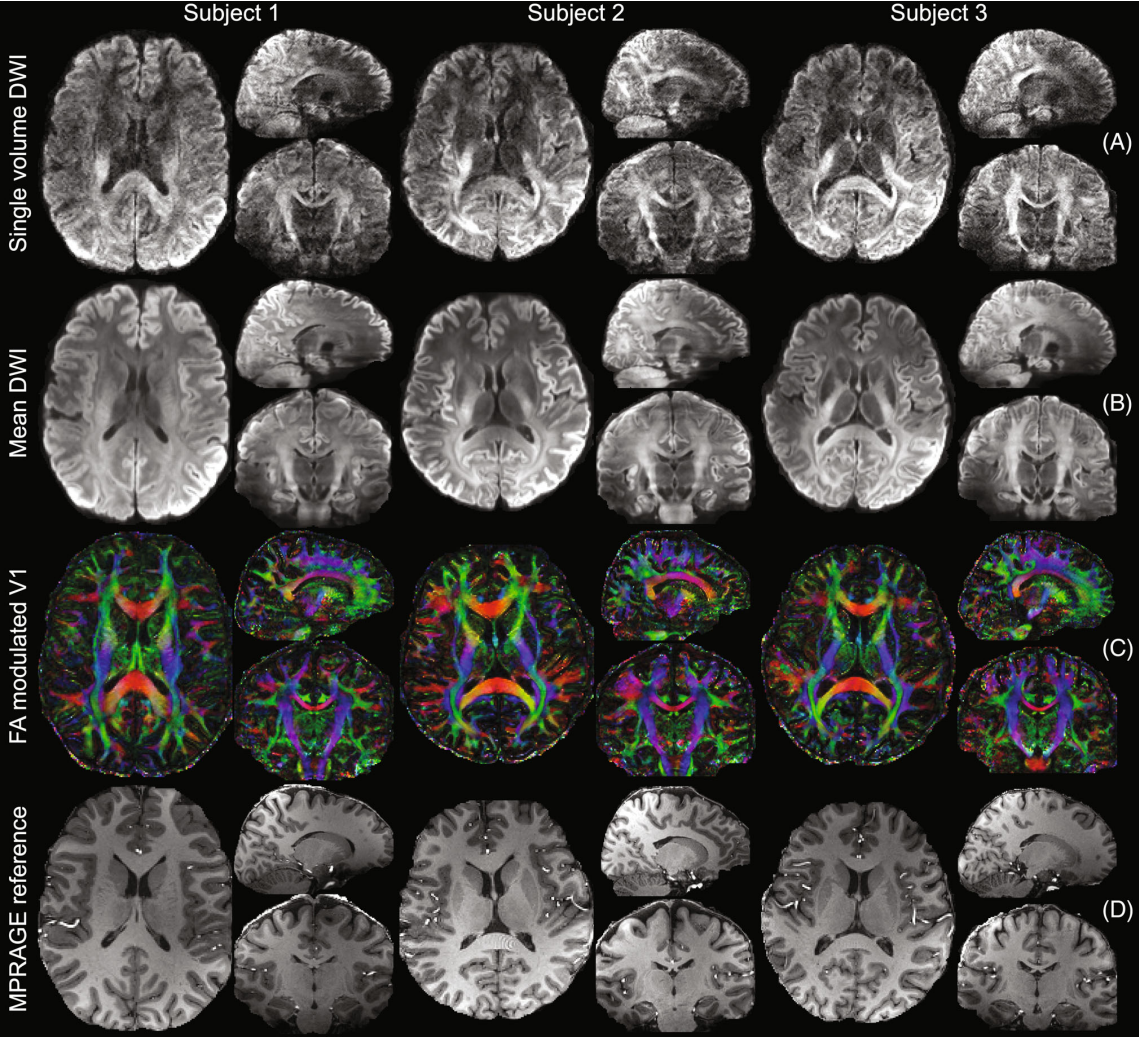
Diffusion MRI results of multiple subjects using the proposed method. The single volume DWI along the diffusion direction (−0.26, −0.81, −0.52) (A), the mean DWI (B), and the fractional anisotropy (FA) modulated primary eigenvector (V1) of diffusion tensor (C), and the anatomical images for reference (D) of three subjects are displayed. The diffusion images are acquired with the high‐resolution protocol at 1.05 mm isotropic resolution using joint blip‐up/down with CAIPI‐PF acquisition. The anatomical images are acquired with MPRAGE at 0.86 mm isotropic resolution and co‐registered to diffusion space for comparison.

These results generalize to other subjects (Figure 9). The single volume diffusion‐weighted images show high‐SNR and sharp textures. The DTI maps demonstrate high‐quality and resolve fine structures with the high‐resolution. The mean DWI results demonstrate high anatomical fidelity, with high structural similarity compared to the anatomical reference. Results of two other subjects are shown in Figure S8.

**FIGURE 10:**
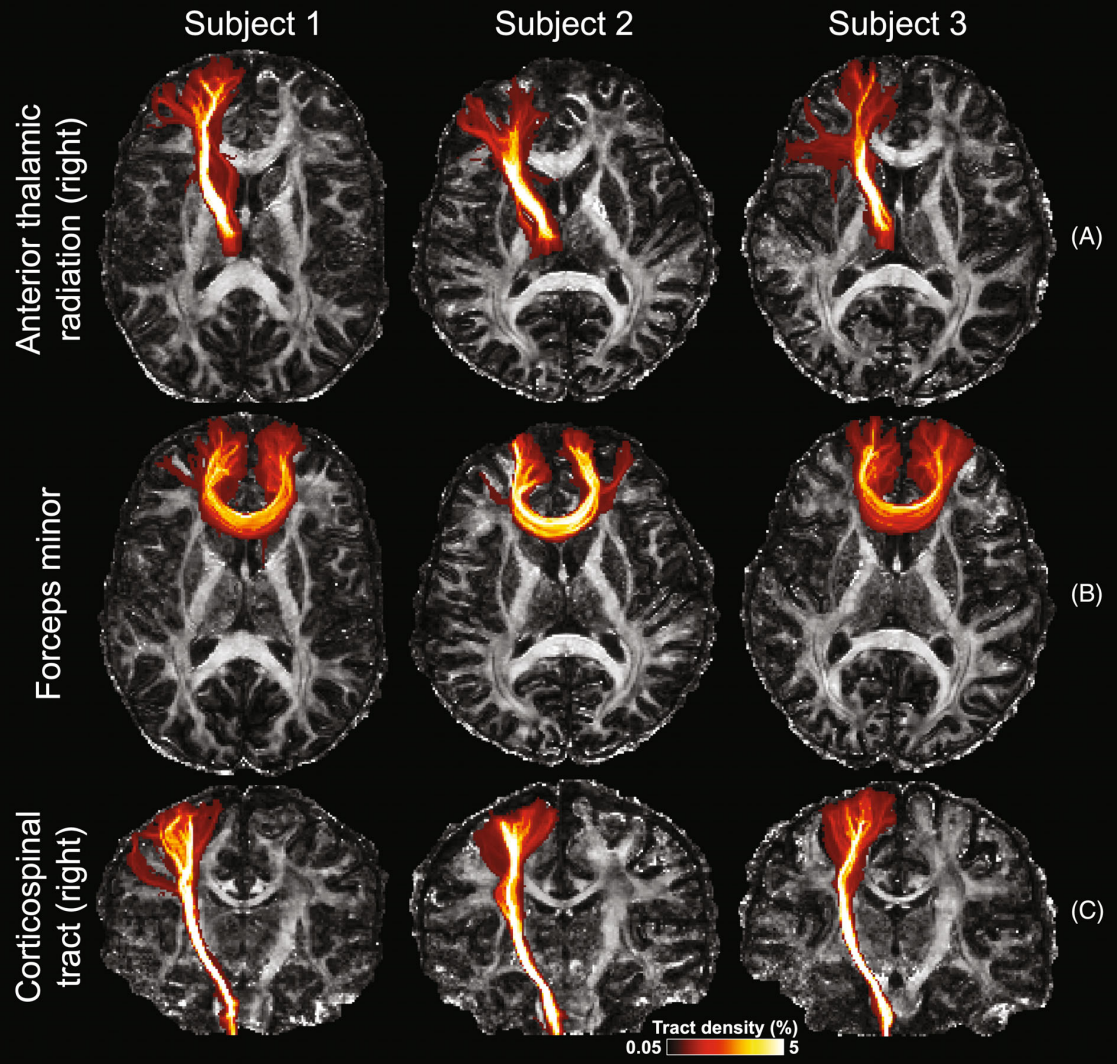
Maximum intensity projections of fiber tracking results from three subjects. Three tracts including right anterior thalamic radiation (A), forceps minor (B), and right corticospinal tract (C) derived from data acquired with the high‐resolution protocol from three subjects using the joint blip‐reversed CAIPI‐PF acquisition are displayed, overlayed on their fractional anisotropy (FA) maps. All tracts are visualized using the same track density threshold (0.05%–5%).

Tractography results for these subjects are shown in Figure 10. The anterior thalamic radiation and forceps minor project successfully into the cortical gray matter in regions of strong field inhomogeneity. The corticospinal tract is well reconstructed even in inferior regions such as the pons where B_0_ field inhomogeneity is strong. The tractography results for these slices without maximum intensity projections are shown in Figure S10.

## 4 DISCUSSION

In this work, we demonstrate that high‐resolution 3D multi‐slab diffusion MRI with minimal distortion and slice aliasing can be achieved by incorporating multiple sampling strategies into a joint reconstruction framework. For accurate distortion correction, both blip‐up and blip‐down shots for each diffusion direction were acquired for field mapping and distortion suppression. Over‐sampling along k_z_ was used for an extended FOV to minimize slice aliasing at slab boundaries. Blip‐reversed based distortion correction and boundary slice aliasing correction have been proposed previously.^9^, ^17^, ^18^, ^19^, ^20^ One of the key challenges in this work is to acquire blip‐reversed data with k_z_ over‐sampling without requiring longer scan time than the conventional 3D multi‐slab diffusion acquisition.

We addressed this challenge using two alterations in the sampling pattern. First, blipped‐CAIPI makes more efficient use of coil sensitivity and improves the conditioning of reconstruction. This is essential for 3D multi‐slab imaging as each slab is very thin and variation of coil sensitivity across the slab is extremely limited. Without CAIPI the reconstruction is highly ill‐posed and lead to high noise amplifications (Figure 4,iii). Blipped‐CAIPI uses the in‐plane coil profiles to encode information, improving the conditioning of the reconstruction and reducing the g‐factor significantly. A relatively large slab thickness (˜20 mm) is used in the high‐resolution protocol. Previous studies demonstrated the 2D navigator corrects most motion‐induced phase errors even for slabs as thick as 30 mm^9^ while the ideal range of slab thickness is 10–20 mm^7–9^ for optimal phase error correction performance. Further evaluations are necessary to determine the optimal slab thickness in this highly accelerated scenario and achieve a well‐balanced compromise between under‐sampled reconstruction and phase correction performance.

Second, our k_z_ partial Fourier strategy enables a denser sampling of the k‐space center compared to regular under‐sampling, which reduces the aliasing artifacts. The improvement is less obvious in stage 1 reconstruction (Figure 4,iv,v), but is substantial in terms of the accuracy of the estimated field map and the image quality of stage 2 joint reconstruction (Figure 5,iv,v). The resultant blurring along the slice direction in stage 1 reconstruction (residual structures in Figure 4B,v) introduced by zero‐filling of the partial Fourier region does not have an obvious impact on the accuracy of the estimated field map, presumably because field maps are intrinsically smooth, especially along the slice direction for thin slabs. In the joint reconstruction, such blurring is reduced thanks to the complementary patterns of blip‐up and blip‐down sampling. Furthermore, to extend the application of our proposed framework to higher spatial resolutions and/or higher b values that pose greater SNR challenges, one could incorporate partial Fourier encoding along the phase‐encoding direction in our framework.

The SPIRiT reconstruction used in this work is a model‐based iterative k‐space reconstruction approach^26^ that outperforms conventional k‐space reconstruction methods such as GRAPPA^31^ by making more efficient use of acquired data. Its iterative self‐consistent formulation provides a noise averaging effect that reduces the overall noise^26^, ^42^ and eliminates the need to train many kernels for our non‐uniform sampling patterns as required by GRAPPA. Additionally, its model‐based formulation allows straightforward incorporation of distortion and phase error correction into the reconstruction, which is essential for our work. Compared to image‐space reconstruction methods such as SENSE,^43^ SPIRiT is advantageous in regions where accurate explicit sensitivity maps are challenging to obtain due to low SNR or subject motion.^44^, ^45^ Our experiments show the SPIRiT constraint improves SNR while preserving the sharp textures (Figure S3). The g‐factors of our reconstruction are also low, probably thanks to the implicit conditioning of noise for k‐space‐based reconstruction^32^ and the noise averaging effect of SPIRiT regularization.^26^ Moreover, our regularized reconstruction framework offers considerable flexibility and can accommodate additional constraints to enhance the reconstruction performance. In our present study, we employed the sparsity constraint to suppress noise. We also made a preliminary attempt to exploit the redundancy across different shots using a structured low‐rank constraint^18^, ^46^ (Figure S11). The use of tailored constraints based on prior knowledge may further boost performance. Furthermore, our reconstruction can be highly parallelized because we used 2D SPIRiT (i.e., separately reconstructing each $k_{y}-k_{z}$ plane) in our framework. Using 3D SPIRiT may potentially further improve the reconstruction by leveraging data redundancy along the readout direction, but at a cost of longer computation time. Future work will be needed to explore the performance of 3D SPIRiT on the 3D multi‐slab data.

The proposed framework's motion robustness can be enhanced by integrating motion correction into the reconstruction process. Although the current in‐vivo results did not exhibit significant head motion, as the scan duration of 36 s for each diffusion direction is similar to the conventional method, it is important to note that head motion might still compromise the multi‐shot joint reconstruction. To address this issue, a promising strategy is to incorporate the motion parameters estimated by “topup” into the forward model of the stage 2 joint reconstruction.^47^

It is worth emphasizing the importance of correcting the slab boundary slice aliasing in our framework. Our stage 2 joint reconstruction relies on an accurate field map to produce distortion‐corrected reconstruction for each slab, which will be affected by boundary slice aliasing in stage 1 reconstruction. Therefore, removing boundary slice aliasing with over‐sampling along kz is essential for both aliasing‐free reconstruction and accurate field map estimation. We utilized “NPEN”^20^ to combine slabs and to reduce saturation artifacts. However, residual artifacts of slab combination still exist (Figures 7 and 9), presumably due to the strong B_0_ field inhomogeneity at 7T that leads to slab profile distortion and shift, violating the assumption of “NPEN” that slab boundary artifacts are periodic along the slice dimension.^20^ Nevertheless, these residual artifacts are much reduced in DTI fitting due to the normalization with the *b* = 0 data. These residual artifacts can be potentially reduced by improving the shimming and by incorporating B_0_ information into the slab profile estimation.

The distortion correction efficacy of the proposed method is largely determined by the accuracy of field map estimation from stage 1 reconstruction. It is helpful to consider the “topup” correction using fully sampled blip‐up and blip‐down data as an upper bound for performance. In extreme cases when distortion is too strong for “topup” to correct even with fully sampled blip‐up and blip‐down data, the proposed method would have limited performance similar to “topup” correction. This is reflected in the results of one subject (Figure S9, Subject 4), where residual distortions in the frontal region remain even after the joint reconstruction, probably due to the extremely high initial distortion level. Nevertheless, the level of residual distortion in the proposed method is highly similar to the “topup” corrected *b* = 0 image with fully k_z_ sampling (Figure S9), indicating that the main source of the residual distortions is the extreme field inhomogeneity, which was likely due to poor shimming for that particular scan. A potential method to improve this is to use additional hardware to provide more homogeneous B_0_ field (e.g., using local shimming coils^18^).

## 5 CONCLUSIONS

In this work, an acquisition and reconstruction framework are developed to minimize the distortion and boundary slice aliasing in high‐resolution 3D multi‐slab diffusion MRI without increasing the scan time. The designed method achieves high‐fidelity, robust reconstruction and produces high‐SNR, high‐quality diffusion images with superior anatomical fidelity compared to the conventional 3D multi‐slab acquisition approach. The value of the method is demonstrated in improving the DTI fitting and tractography and can be further explored in more types of applications and by pushing to higher, submillimeter isotropic resolutions.

## Supporting information


**Data S1.** Supporting information.

## Data Availability

In support of Magnetic Resonance in Medicine's reproducible research goal, the MatLab code used for the reconstructions performed in this study is available at https://github.com/liziyu0929/distortion‐free‐3d‐diffusion‐mri.
