# Supplementary material for: Sampling strategies and integrated reconstruction for reducing distortion and boundary slice aliasing in high‐resolution 3D diffusion MRI
Source: Magn Reson Med. 2023 Jun 15;90(4):1484–501. doi: 10.1002/mrm.29741 (PMC10952965; doi:10.1002/mrm.29741)
Supplement: Supplementary file 1 — Data S1. Supporting information. [file MRM-90-1484-s001.docx]

**Sampling strategies and integrated reconstruction for reducing distortion and boundary slice aliasing in high-resolution 3D diffusion MRI**

Ziyu Li^1^, Karla L. Miller^1^, Jesper L.R. Andersson^1^, Jieying Zhang^2^, Simin Liu^2^, Hua Guo^2^, Wenchuan Wu^1*^

^1^Wellcome Centre for Integrative Neuroimaging, FMRIB, Nuffield Department of Clinical Neurosciences, University of Oxford, Oxford, United Kingdom;

^2^Center for Biomedical Imaging Research, Department of Biomedical Engineering, School of Medicine, Tsinghua University, Beijing, China.

^*^Correspondence to: Wenchuan Wu, Ph.D., Wellcome Centre for Integrative Neuroimaging (WIN), FMRIB, John Radcliffe Hospital, Oxford, OX3 9DU, UK. E-mail: wenchuan.wu@ndcn.ox.ac.uk.

**Supplementary Information**

Co-registration Details

Steps for co-registering the CAIPI-PF and conventional 3D multi-slab data with the MPRAGE image:

1. An intermediate b=0 image volume (denoted as $I_{1}$) was acquired in the same scan as the MPRAGE image (with matched gradient distortions) using conventional 2D EPI and corrected for distortion using FSL’s “topup”^1, 2^ to minimize the impact of gradient nonlinearity distortions and improve the co-registration accuracy.
2. The b=0 images of CAIPI-PF and conventional 3D multi-slab sampling (denoted as $B0_{CAIPI-PF}$ and $B0_{conventional}$) were co-registered to $I_{1}$ using FSL’s “flirt”^3, 4^ with default parameters to correct gradient nonlinearity distortions. The resultant images and transformations were denoted as $I_{2,CAIPI-PF}, I_{2,conventional}$ and $T_{2,CAIPI-PF},T_{2,conventional}$.
3. $I_{2,CAIPI-PF}$ and $I_{2,conventional}$ were co-registered to the MPRAGE image using FSL’s “epi_reg”^3, 4^. The resultant transformations were denoted as $T_{3,CAIPI-PF}$and $T_{3,conventional}$.
4. $T_{2,CAIPI-PF}$ and $T_{2,conventional}$ were combined with $T_{3,CAIPI-PF}$and $T_{3,conventional}$ using FSL’s “convert_xfm”^3, 4^. The resultant transformations were denoted as $T_{4,CAIPI-PF}$and $T_{4,conventional}$.
5. $B0_{CAIPI-PF}$ and $B0_{conventional}$ were co-registered to the MPRAGE image using “flirt” with “bbr” as the cost function^5^ and $T_{4,CAIPI-PF},T_{4,conventional}$ as initializations. The resultant transformations were used to resample the results from the diffusion analyses into the anatomical space.


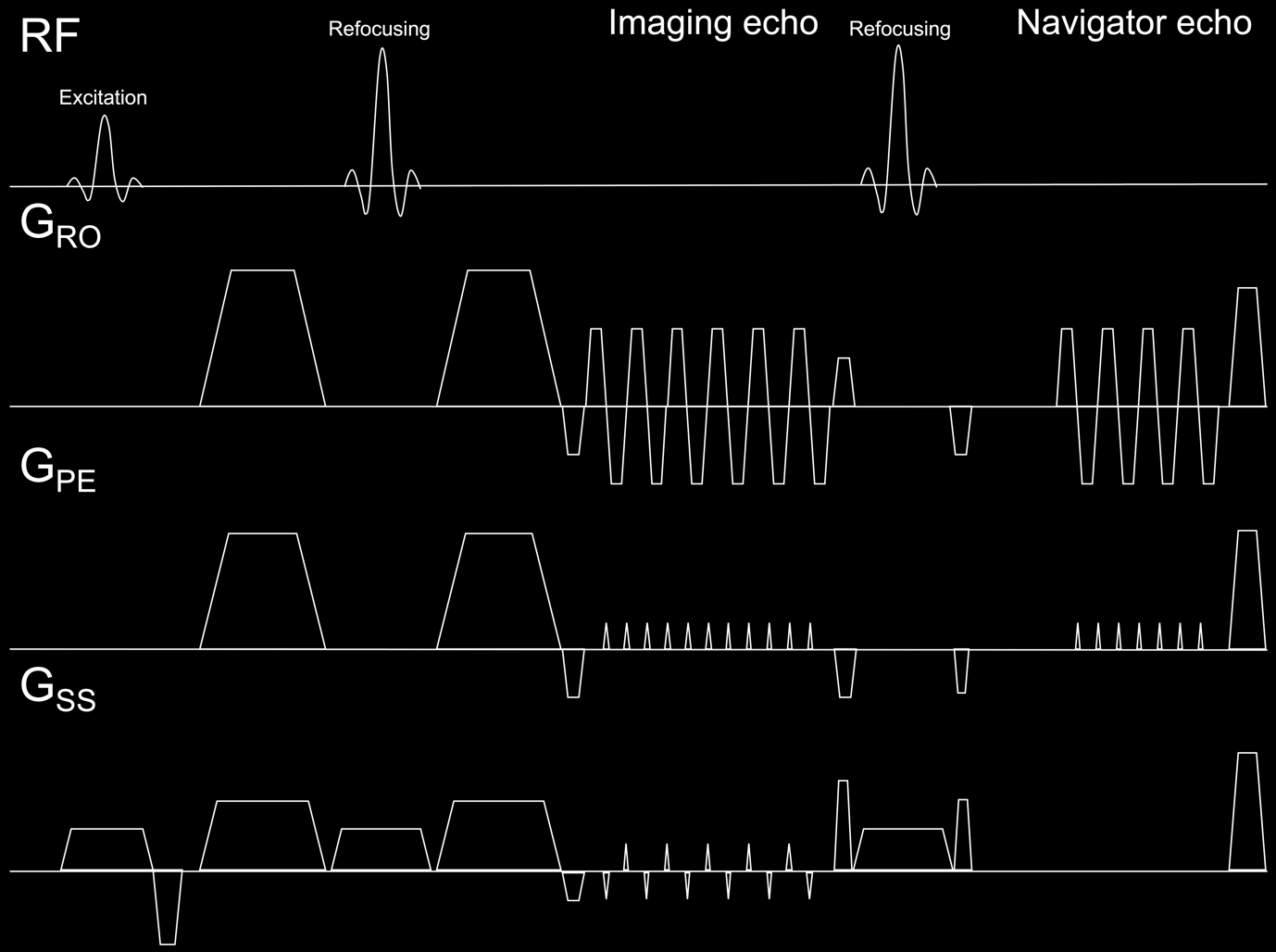


**Figure S1. Sequence diagram of the proposed 3D multi-slab CAIPI-PF acquisition.** The diagrams of the RF pulse, the readout gradient (G_RO_), the phase-encoding gradient (G_PE_), and the slice selection gradient (G_SS_) are displayed. The kz-blipped CAIPI is achieved by adding slice selection blipped gradient. The 3D imaging echo is followed by a navigator echo used for 2D navigator acquisition.


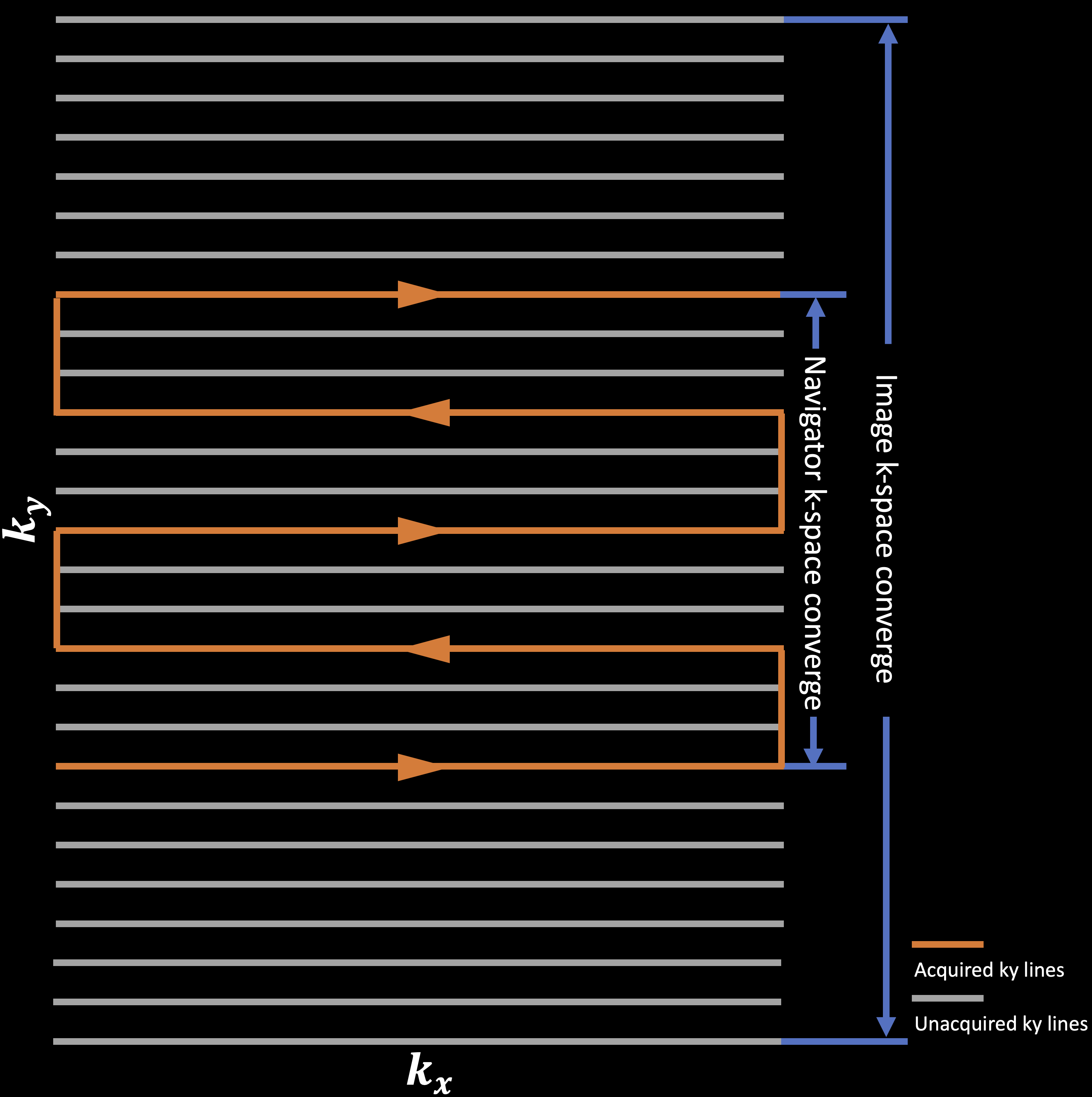


**Figure S2. Illustration of 2D navigator acquisition trajectory.** The 2D navigator acquires the central kz plane of each slab after the imaging echo. Only the central part of the 2D k-space (i.e., 64 phase-encoding lines) is acquired with the same phase-encoding direction, the same ky under-sampling factor (R_y_=3), and the same echo spacing as the imaging echo.


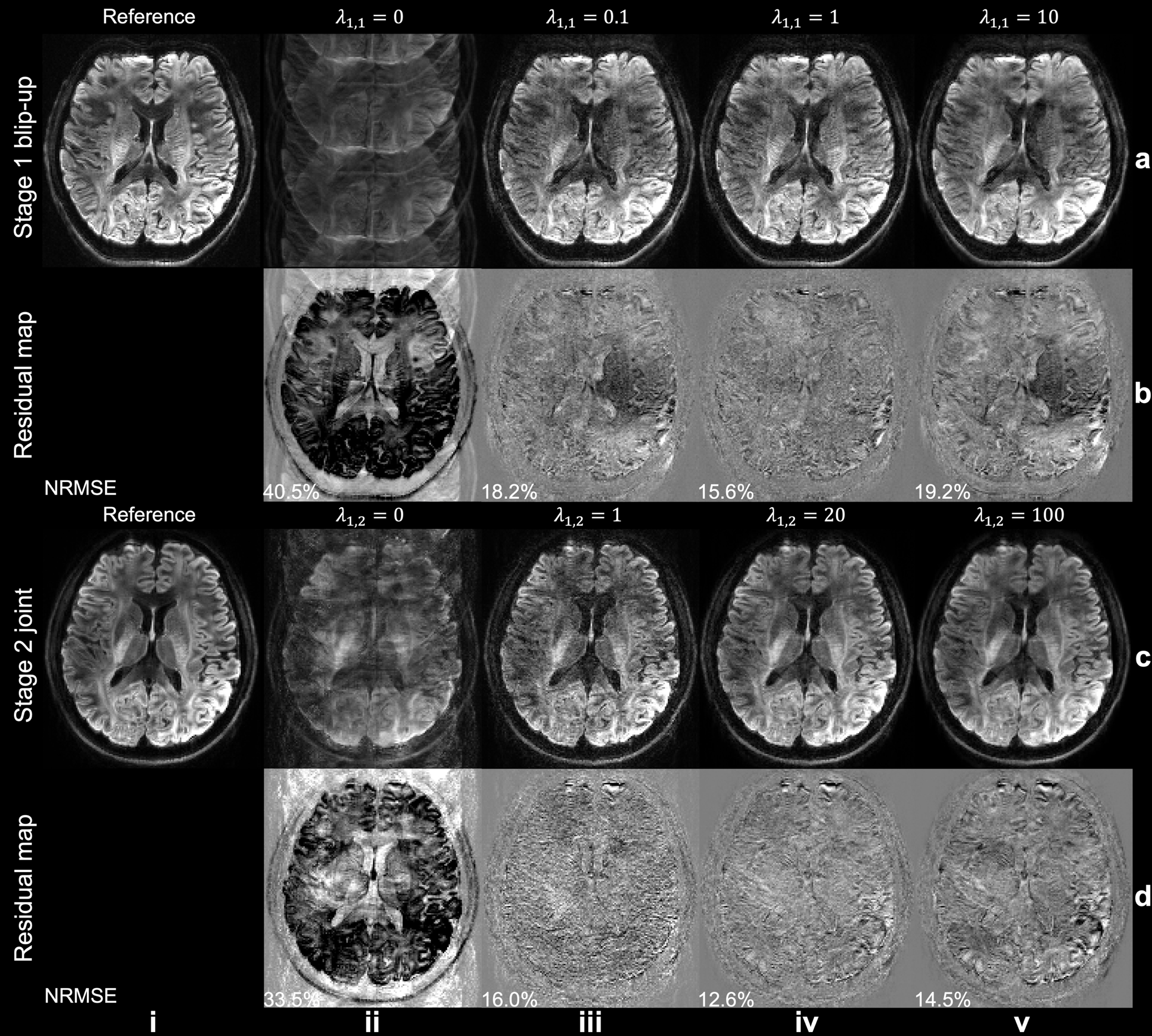


**Figure S3. Impact of SPIRiT regularization weight on reconstruction.** Stage 1 blip-up (a) and stage 2 joint reconstruction (c) of CAIPI-PF data using different SPIRiT regularization weights and their residuals compared to the reference (b, d) acquired with the evaluation protocol (1.22 mm isotropic resolution) are displayed. The optimal weight for stage 1 (i.e., $\lambda_{1,1}=1$) was used when different weights were evaluated for stage 2. The normalized root mean squared errors (NRMSE) of the whole slab are listed to quantify the image similarity.


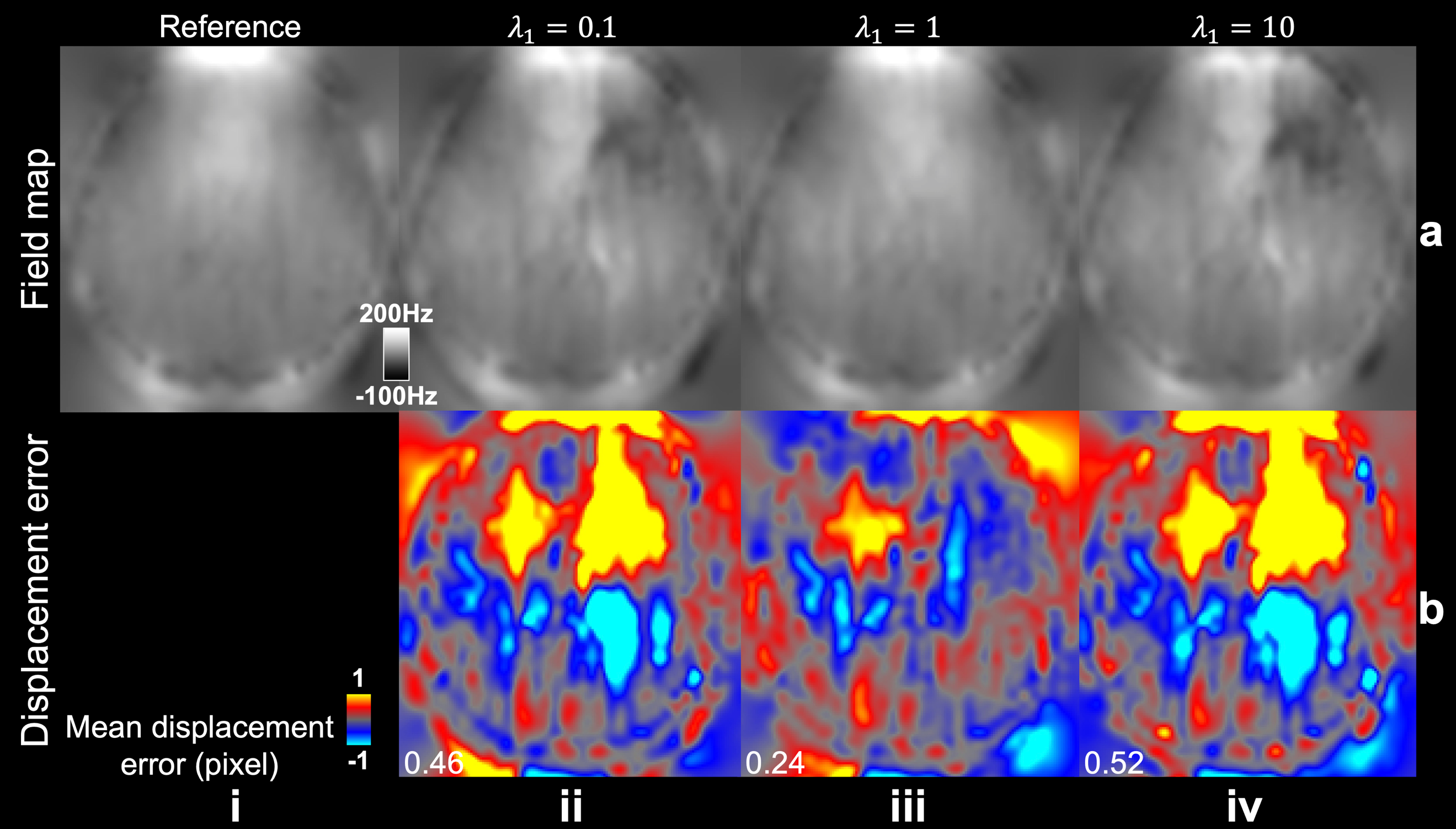


**Figure S4. Impact of stage 1 SPIRiT weight on field map estimation.** The field maps from “topup” estimated using reference blip-up/down data (a, i) and CAIPI-PF blip-up/down data with different SPIRiT weights for stage 1 reconstruction (a, ii-iv) and their voxel displacement error maps with the reference field map (b) acquired with the evaluation protocol (1.22 mm isotropic resolution) are displayed. The mean displacement errors of the whole slab are listed to quantify the image similarity.

**
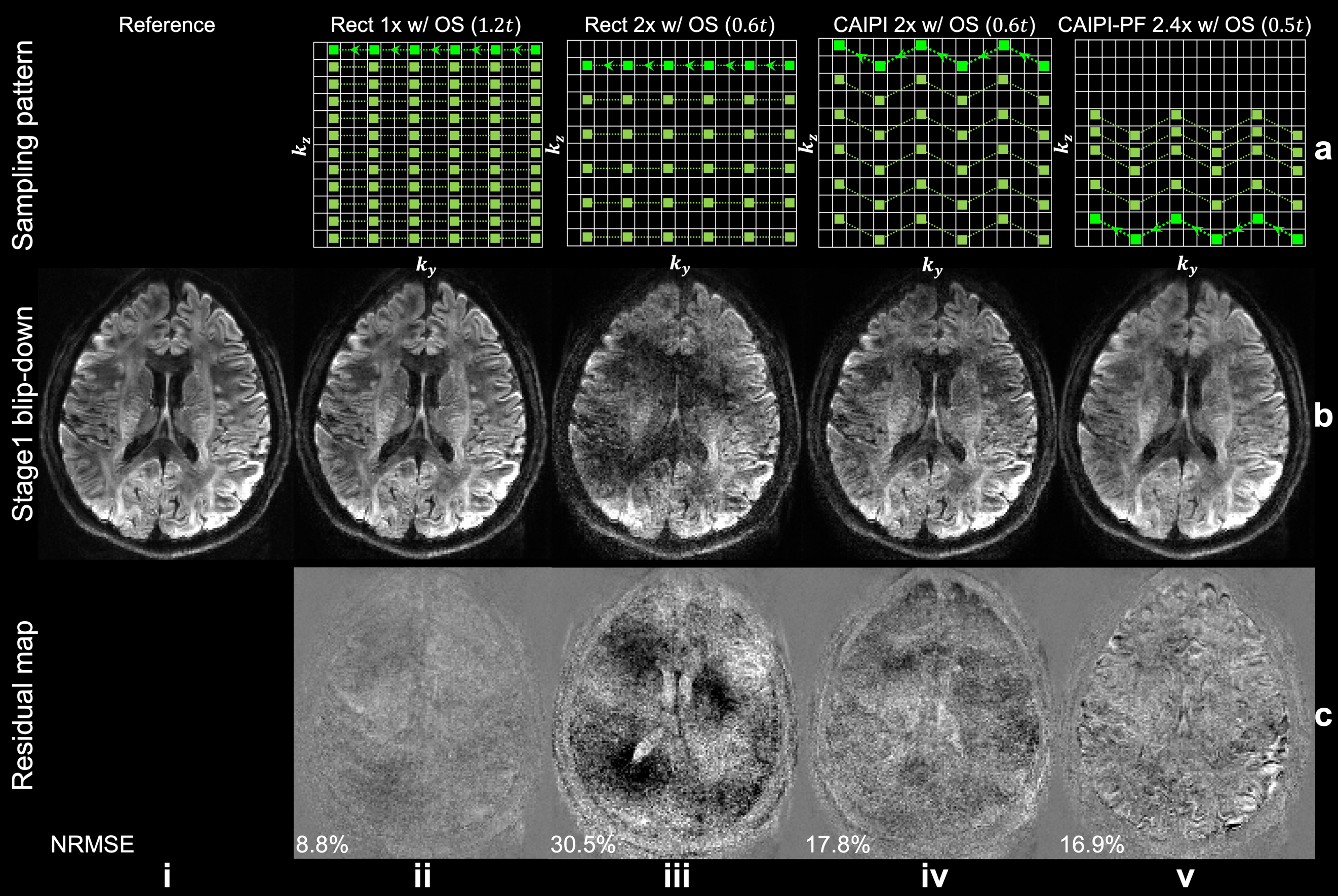
**

**Figure S5. Stage 1 blip-down sampling and reconstruction.** The sampling patterns (a), reconstruction results of a slab-central slice (b) from fully sampled reference (i) and different sampling patterns (ii-v) and their residuals with the fully sampled reference (c) for the blip-down data acquired with the evaluation protocol (1.22 mm isotropic resolution) are displayed, with relative scan times listed for each method. The trajectory of one shot of the multi-shot sampling is marked in bright green with arrows. The normalized root mean squared errors (NRMSE) of the whole slab are listed to quantify the image similarity. The parameter $t$ represents the scan time of a rectangular sampling without over-sampling or acceleration along kz (as Fig. 2c, i).


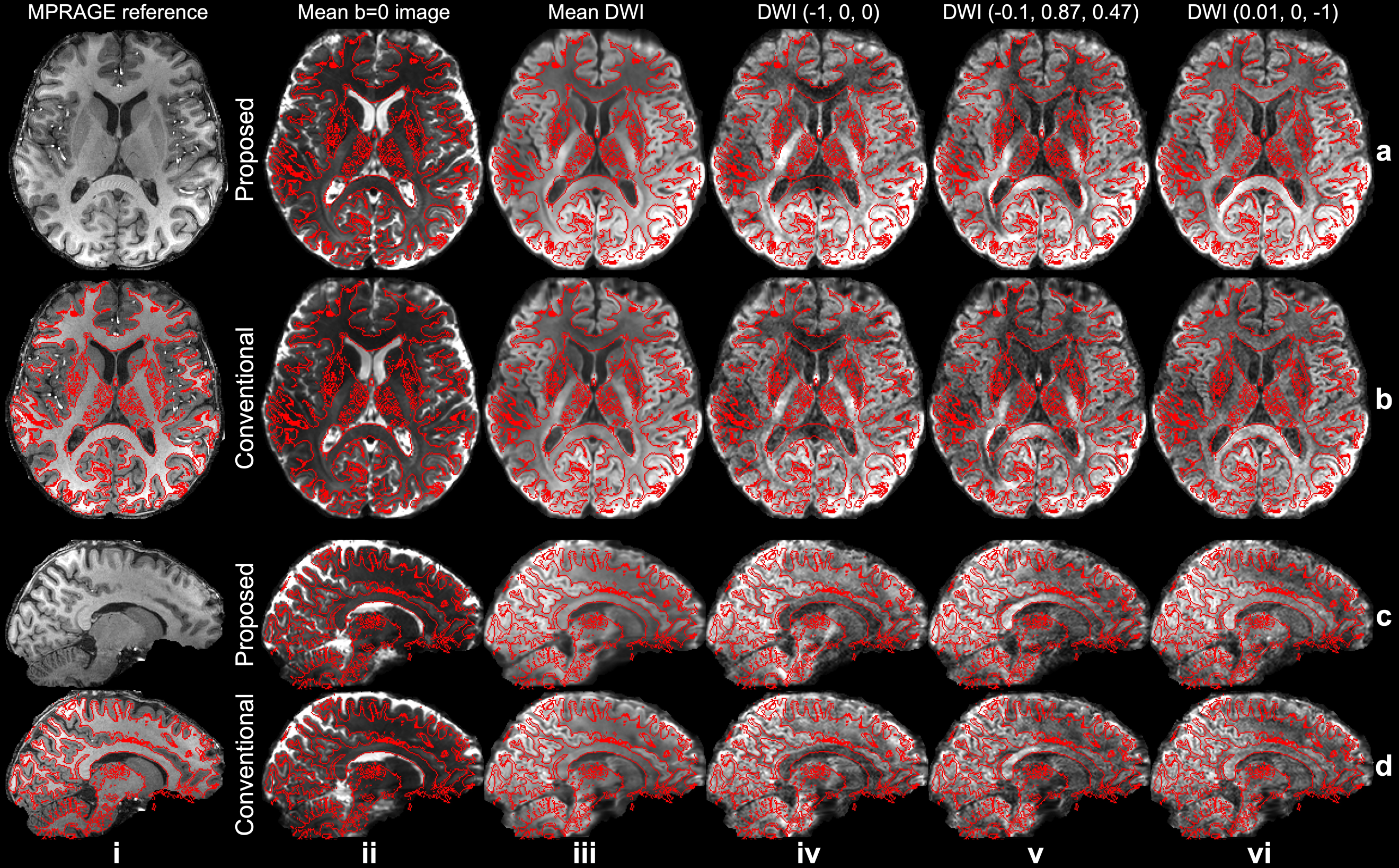


**Figure S6. Comparison of image anatomical fidelity.** The axial (a, b) and sagittal (c, d) views of the MPRAGE reference (0.86 mm isotropic resolution, i), mean b=0 images (ii), mean diffusion-weighted images (DWI) (iii), and three DWI along different diffusion directions (iv-vi) acquired with CAIPI-PF sampling (Proposed, a, c) and conventional 3D multi-slab sampling (Conventional, b, d) at 1.05 mm isotropic resolution from the same subject are displayed. The write matter boundary segmented by FSL’s “fast” is marked in red and overlayed. The diffusion data are co-registered to the MPRAGE reference for comparison.


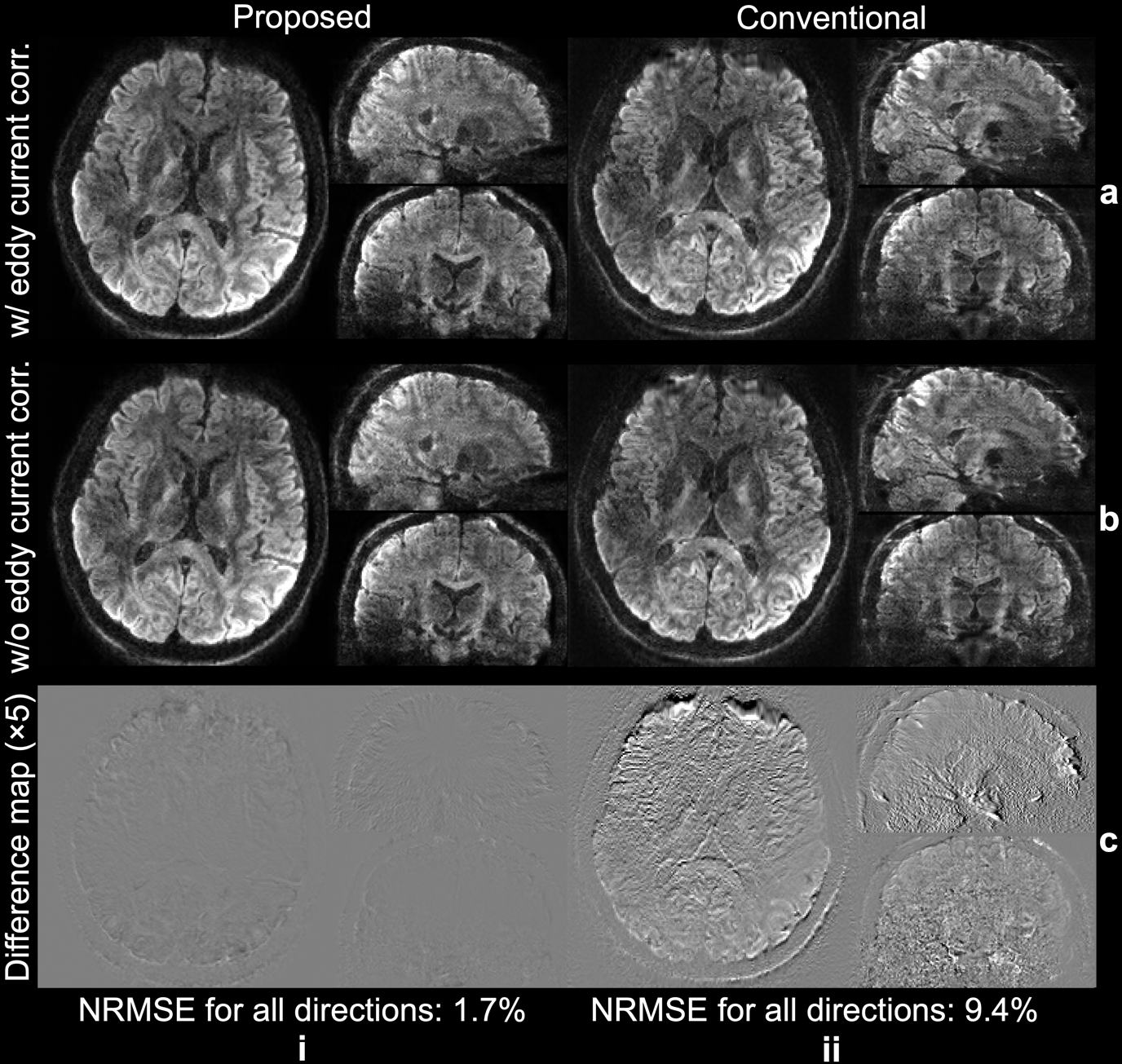


**Figure S7. Correction of eddy current induced distortion.** The single volume diffusion-weighted image along diffusion direction (-0.16, -0.63, 0.76) acquired with CAIPI-PF (Proposed, i) and conventional 3D multi-slab sampling (Conventional, ii) at 1.05 mm isotropic resolution processed by FSL’s “eddy” with eddy current correction (a), without eddy current correction (b), and their difference maps (c) of the same subject are displayed. The normalized root mean squared errors (NRMSE) for diffusion-weighted images of all diffusion directions are listed to quantify the image similarity.

**
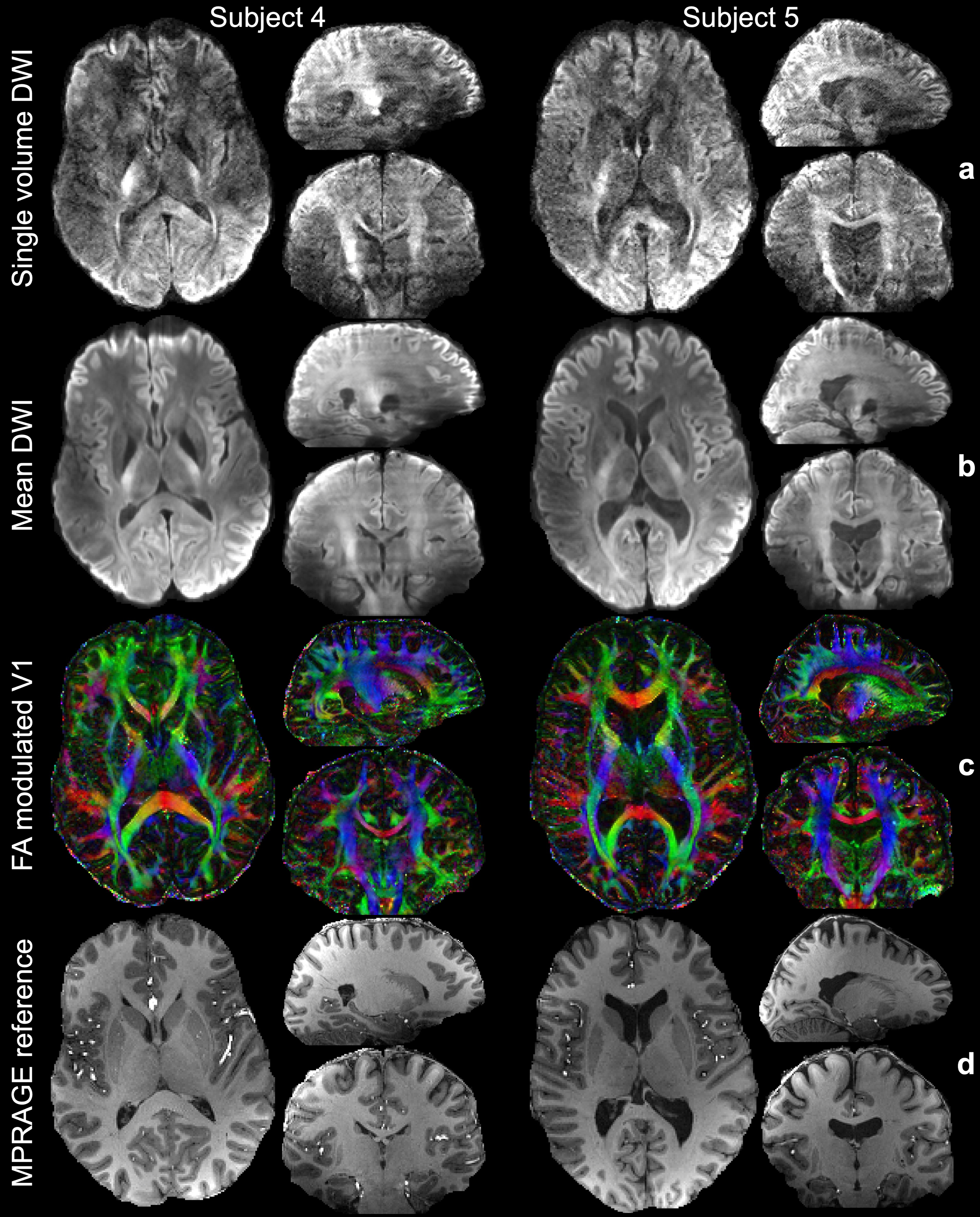
**

**Figure S8. Diffusion image results of the remaining subjects.** The single volume diffusion-weighted images (DWI) along (-0.26, -0.81, -0.52), the mean DWI (b), and the fractional anisotropy (FA) modulated primary eigenvector (V1) of diffusion tensor (c), and the anatomical images for reference (d) of two subjects are displayed. The diffusion images are acquired with CAIPI-PF sampling at 1.05 mm isotropic resolution. The anatomical images are acquired with MPRAGE at 0.86 mm iso. resolution and co-registered to diffusion images for comparison.


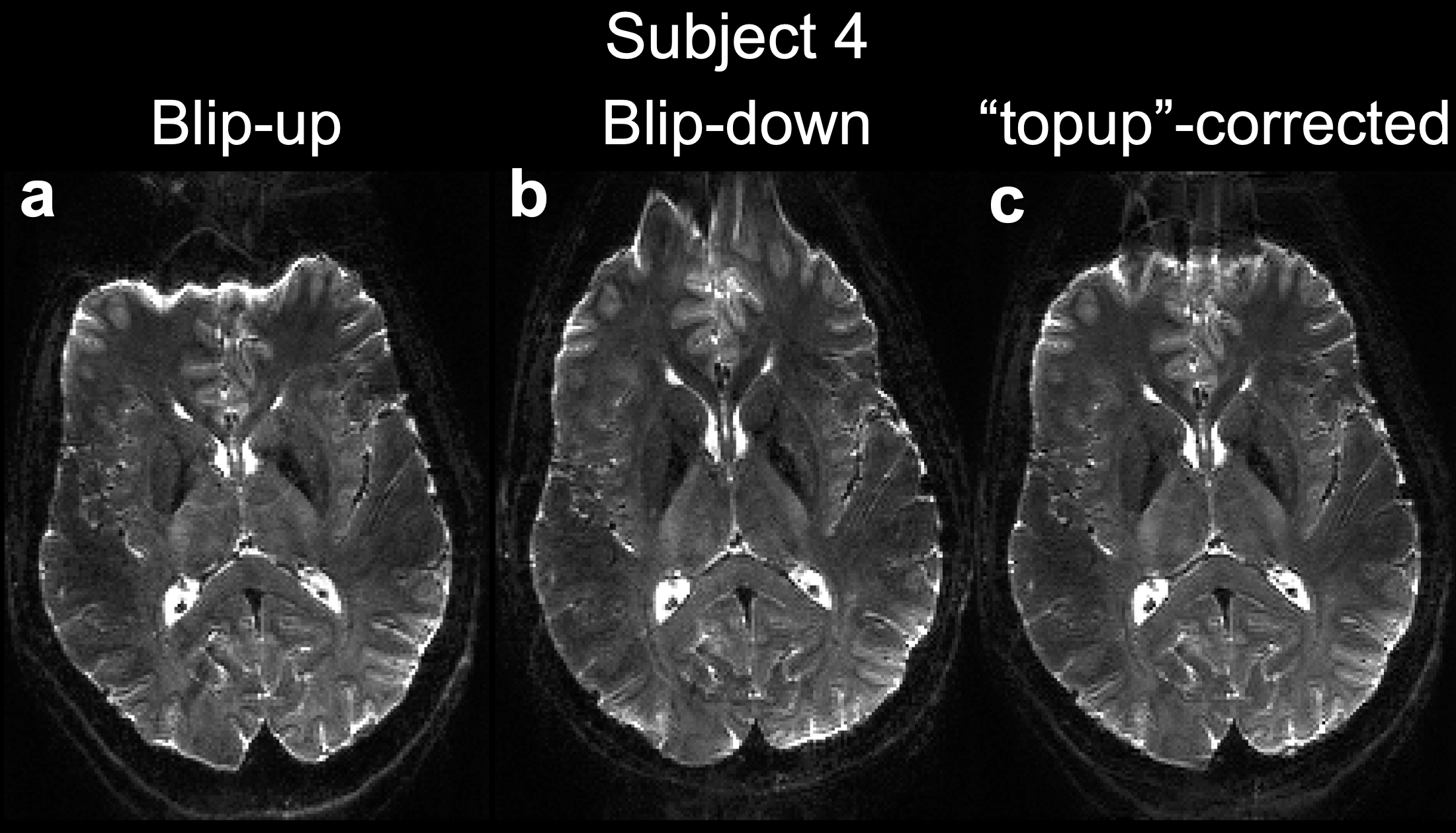


**Figure S9. The b=0 images of a representative subject.** An axial slice of blip-up (a), blip-down (b), and “topup”-corrected (c) b=0 images of a representative subject scanned with CAIPI-PF sampling at 1.05 mm isotropic resolution are displayed.

|  | **Proposed (mm^3^)** | **Conventional (mm^3^)** | **Intersection (mm^3^)** |
| --- | --- | --- | --- |
| **Right anterior thalamic radiation** | 8955.4 | 7448.2 | 5205.8 |
| **Forceps minor** | 14281.6 | 9990.3 | 8017.7 |
| **Right corticospinal tract** | 11479.0 | 7679.7 | 6328.7 |

**Table S1. Tract mask volumes.** Tractography results of data from proposed CAIPI-PF (Proposed) and conventional 3D multi-slab (Conventional) sampling are binarized to obtain tract masks for different tracts (threshold: 0.3%). Tract mask volumes are computed for tract masks of proposed and conventional data and their intersections.

**
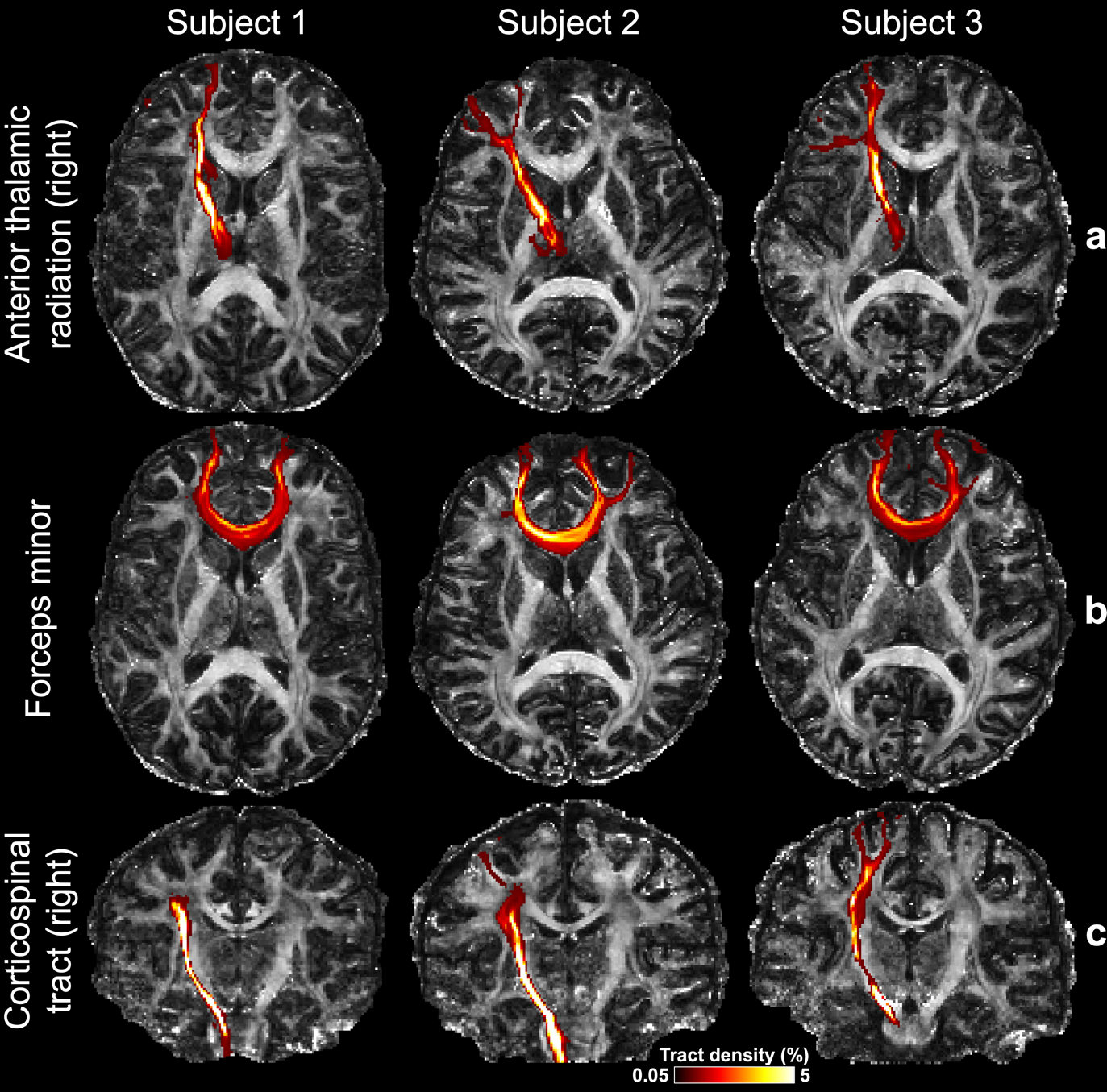
**

**Figure S10. Tractography of multiple subjects.** Tractography results (not maximum intensity projected) of three tracts including right anterior thalamic radiation (a), forceps minor (b), and right corticospinal tract (c) with tract density range 0.05%-5% from three subjects using the CAIPI-PF sampling (1.05 mm isotropic resolution) overlayed on their fractional anisotropy (FA) maps are displayed.


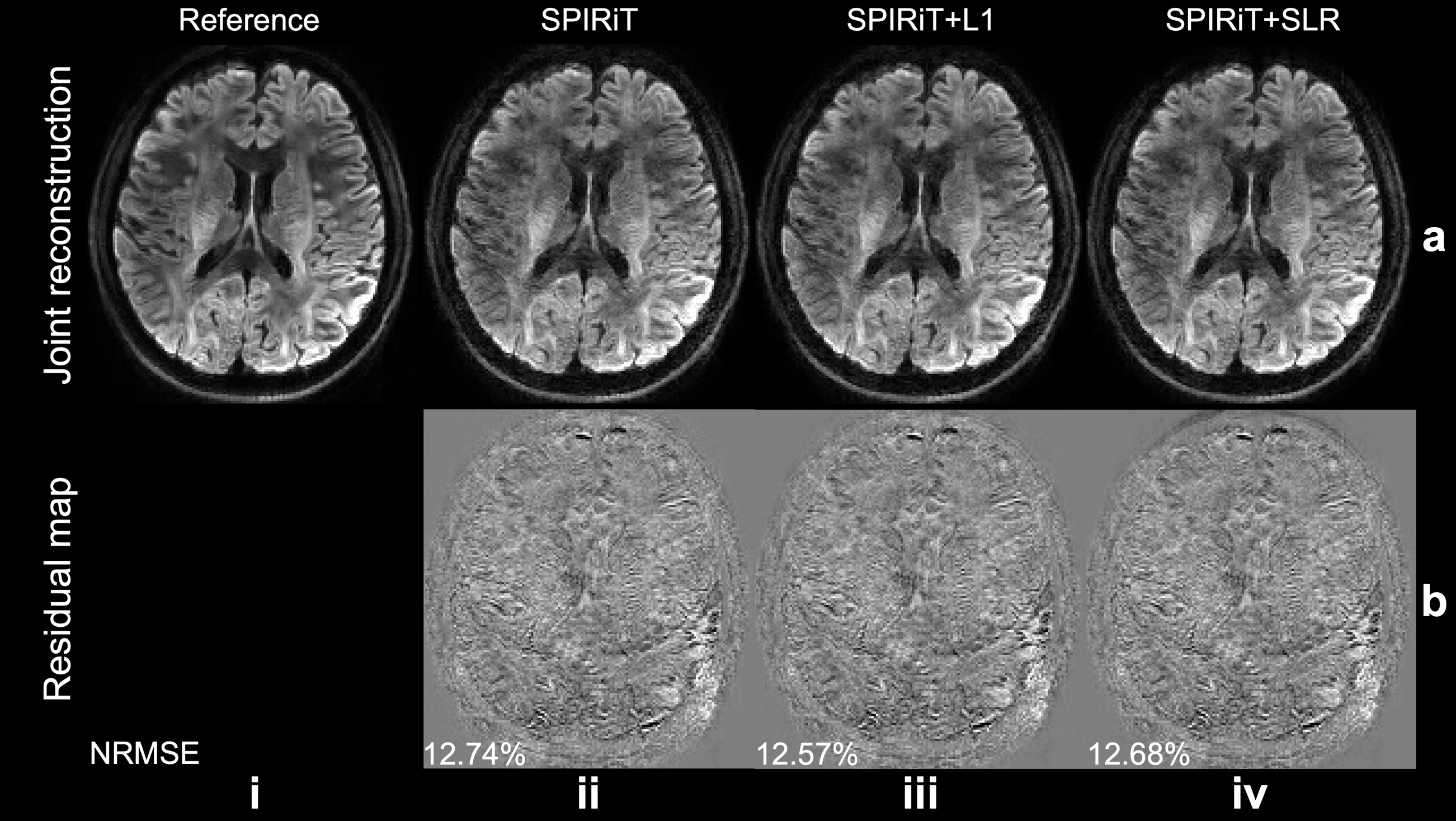


**Figure S11. Comparison of different reconstruction methods.** Stage 2 joint reconstruction (a) of fully sampled reference data (i) and under-sampled CAIPI-PF data (Ry/Rz=3/2.4) using different reconstruction methods (SPIRiT (ii), SPIRiT + L1 (iii), and SPIRiT + structured low-rank (SLR) (iv)) and their residuals compared to the reference (b) acquired with the evaluation protocol (1.22 mm isotropic resolution) are displayed. The normalized root mean squared errors (NRMSE) of the whole slab are listed to quantify the image similarity.

**Reference:**

1. Andersson JL, Skare S, Ashburner J. How to correct susceptibility distortions in spin-echo echo-planar images: application to diffusion tensor imaging. *Neuroimage*. 2003;20(2):870-888.

2. Smith SM, Jenkinson M, Woolrich MW, et al. Advances in functional and structural MR image analysis and implementation as FSL. *Neuroimage*. 2004;23:S208-S219.

3. Jenkinson M, Smith S. A global optimisation method for robust affine registration of brain images. *Medical image analysis*. 2001;5(2):143-156.

4. Jenkinson M, Bannister P, Brady M, Smith S. Improved optimization for the robust and accurate linear registration and motion correction of brain images. *Neuroimage*. 2002;17(2):825-841.

5. Greve DN, Fischl B. Accurate and robust brain image alignment using boundary-based registration. *Neuroimage*. 2009;48(1):63-72.
